# Flexural Behavior of Structural Foam Concrete with Hollow Polymer Spherical Fillers Under Thermal and Mechanical Loading: Numerical and Experimental Analysis

**DOI:** 10.3390/polym18202580

**Published:** 2026-10-20

**Authors:** Alexey N. Beskopylny, Ivan Panfilov, Diana M. Shakhalieva, Natalya Shcherban’, Sergey A. Stel’makh, Evgenii M. Shcherban’, Andrei Chernil’nik

**Affiliations:** 1 Department of Transport Systems, Faculty of Roads and Transport Systems, Don State Technical University, 344003 Rostov-on-Don, Russia; 2 Department of Theoretical and Applied Mechanics, Agribusiness Faculty, Don State Technical University, Gagarin Square, 344003 Rostov-on-Don, Russia; panfilov.i@gs.donstu.ru; 3 Department of Design, Don State Technical University, 344003 Rostov-on-Don, Russia; delshaeva@donstu.ru; 4 Department of Unique Buildings and Constructions Engineering, Don State Technical University, 344003 Rostov-on-Don, Russia; ndocenko@donstu.ru (N.S.); sstelmah@donstu.ru (S.A.S.); achernilnik@donstu.ru (A.C.); 5 Department of Engineering Geometry and Computer Graphics, Don State Technical University, 344003 Rostov-on-Don, Russia; etsherban@donstu.ru

**Keywords:** foam concrete, polymer-composite, polymers, polypropylene, polyamide, thermal conductivity, flexural strength

## Abstract

Creating low-density composite materials that can handle significant temperature changes and bending stress is a promising approach for building floating platforms. This study creates a numerical model and conducts an experimental analysis of foam concrete containing polymer spheres with different diameters and properties. Laboratory bending tests were performed on manufactured foam concrete samples containing 10 mm, 12 mm, 15 mm, 19.05 mm, and 20 mm diameter polypropylene spheres. To predict the thermophysical properties of a composite featuring structural foam concrete with embedded polymer spheres, a numerical model was developed in the Material Designer module as the first step. The resulting density, thermal conductivity, and specific heat dependences were used as input for subsequent numerical analysis in ANSYS under nonuniform temperature and bending loading, based on the nonlinear Menetrey–Willam model. By comparing load–displacement curves for different sphere sizes, the accuracy of the model in forecasting strength and deformation properties was established. The study revealed that the maximum equivalent von Mises stress was 3.3 MPa, indicating that the material operates in the elastic zone, but close to crack formation. The maximum displacement due to temperature differences was 0.087 mm, and the tensile stress in the longitudinal direction was σ_X_ = 0.18 MPa. This indicates that the composite performs satisfactorily under temperature differences from +4 °C to −60 °C. Patterns of failure of structural foam concrete specimens with polymer spheres under bending were analyzed, demonstrating that cracking starts in the lower chord of the tensile zone, weakened by the presence of the spheres.

## 1. Introduction

The creation of hollow concrete slabs with polymer spheres is a new technology that reduces the weight of structures, improves sound and thermal insulation, and creates channels for utility lines [1,2]. In recent years, this technology has been used to create floating platforms for energy, oil, and gas production facilities, representing a promising new type of construction [2,3,4,5]. Key aspects of their application include weight reduction due to internal voids, the use of hydraulic concrete, and the integration of aggregates for buoyancy. The presence of voids or lightweight aggregates (expanded polystyrene, expanded clay concrete) reduces the dead weight of the reinforced concrete structure while maintaining the required buoyancy [6,7,8]. The process can be organized both in a factory setting and directly on the construction site; however, a full recognition of the performance of hollow concrete slabs requires a series of experimental and theoretical studies. Comparative studies of hollow core slabs and conventional flat slabs to examine the behavior of bending moments, shear forces, deflections, and span reactions under various loading conditions clearly demonstrate that hollow core slabs are more effective than conventional flat slabs in terms of strength and cost effectiveness [9,10]. However, the extent of this effectiveness has not been adequately studied and represents a gap in existing research. The problem of modeling the compression of concrete prisms with the addition of hollow polypropylene fillers has been examined in [11]. It is noted that hollow polypropylene spheres significantly reduce the mass of the structure, but also reduce the strength properties of the structural elements. The article notes that to understand the behavior of such elements under bending for the development of floating platforms at sea, a series of new studies taking into account specific loads is necessary. A study on the autogenous shrinkage of high-strength lightweight aggregate concrete demonstrated that the initial water content of the lightweight aggregate plays a crucial role in mitigating autogenous shrinkage. Higher water content facilitates prolonged internal curing and reduces effective autogenous shrinkage by up to 50.6%, while the developed autogenous shrinkage model exhibited good predictive capability [12]. A micromechanical model for predicting the nonlinear, quasi-brittle behavior of expanded-clay lightweight concrete under multiaxial loading enables a detailed description of damage initiation and propagation within the cement matrix [13]. Modeling the flexural behavior of a single-span slab made of continuous-fiber-reinforced lightweight concrete at both ambient and elevated temperatures revealed that moment redistribution toward the deflection zone positively influences the slab’s fire resistance and structural performance under fire exposure [14]. Modeling the flexural behavior of structural foam concrete with hollow polypropylene spherical fillers is a complex task that requires consideration of multiple factors, from the material’s microstructure to numerical analysis methods [15,16,17,18]. The following aspects can be considered when modeling foam concrete with hollow spheres.

Hollow air spheres, which have a random geometry, significantly affect the structure of foam concrete [19]. They create additional pores, reducing the average density of the material, but their presence also changes the nature of crack formation. A key question is how the spheres are distributed within the matrix and how they interact with the cement stone and air cells [20]. This issue has not been sufficiently studied, since modeling the porous structure of concrete in numerical models is too high-dimensional and requires extensive computational resources.

Both analytical and numerical approaches are used to analyze flexural behavior, the most popular of which is the finite element method (FEM) [21,22,23,24]. This method allows for the construction of a computational model of a specimen (beam) taking into account material heterogeneity, pores, and the presence of spheres. The following parameters are input into software packages (ANSYS, ABAQUS): the elastic modulus of the cement stone and spheres, Poisson’s ratio, and the volume fraction of spheres. For further calculations, effective medium methods can be used, which take into account the averaged properties of the composite, simplifying the calculations while maintaining their suitability for engineering problems. At the same time, the nonlinear behavior of concrete and early age cracking lead to significant errors [25,26,27,28]. For a reliable analysis of the numerical modeling results, it is necessary to use experimental calibrations. Numerical models are verified using laboratory bending tests (e.g., three-point bending), followed by a comparison of experimental and calculated values of deflection, curvature, and stress distribution [29,30,31].

The incorporation of hollow spheres into the composite significantly affects its properties. Compared to traditional fibers (e.g., polypropylene), macro spheres offer a number of advantages [32]. A decrease in the foam concrete density leads to a controlled loss of strength. Topological optimization of the structure is another gap and is of great interest to researchers. An example of the topological optimization of a reinforced concrete beam with rectangular and triangular prismatic voids was previously examined. The authors demonstrated a 10.8% reduction in CO_2_ emissions [33]. However, rectangular or triangular void shapes lead to stress concentrations at the corners of the embedded prisms, which undoubtedly reduces load-bearing capacity.

The study of thermophysical properties plays a crucial role in Arctic conditions, where temperature fluctuations are large and lead to an uneven temperature field within the structure. A thermal analysis of massive reinforced concrete structures with a porous structure is presented in [34,35,36]. It is shown that the size and nature of the pore arrangement play a key role in assessing the thermal conductivity coefficient and determining the thermophysical properties of the structure. An uneven thermal field leads to bending of the structure. The effect of macro voids on flexural tensile strength is ambiguous. On the one hand, they can partially compensate for the low tensile strength of cement stone. On the other hand, their spherical shape and low density can lead to the formation of crack “bridges” at the boundary with spheres, which reduces the effectiveness of reinforcement.

The most important tasks in the production of composites with hollow spherical inclusions are the selection of optimal compositional and processing parameters [37,38,39,40,41,42]. The modeling results are significantly affected by volume fraction of micropores and hollow spheres. There is an optimal range (usually 10–30% of the concrete volume), beyond which the effect of reinforcement is leveled out, and the strength can be significantly reduced due to disruption of the matrix continuity. Abdulridha et al. [43] investigated the flexural strength of voided reinforced concrete beams using different truss configurations. The result indicated that slabs with 50 mm voids retained 96.2% of the load-bearing capacity of a solid slab, whereas slabs with 60 mm voids achieved only 86.5%. Dai et al. [44] investigated the dynamic compressive properties of novel reinforced concrete composites incorporating hollow metal spheres. The addition of hollow metal spheres to a concrete matrix in amounts ranging from 10% to 30% increased dynamic strength and total energy absorption by 25.4% and 317%, respectively, at a strain rate of approximately 70 s^−1^. Duan et al. [45] investigated the effect of glazed hollow spheres and dispersed reinforcement on the properties of lightweight ultra-high-strength concrete. Incorporating glazed hollow spheres at a volume fraction of up to 40%, combined with hybrid reinforcement using steel and polypropylene fibers, ensures an optimal balance of density and strength in the production of high-performance lightweight concretes. Weng et al. [46] developed a series of lightweight, ultra-high-strength composite materials using glass spheres. A 30% content of glass microspheres in a lightweight, ultra-high-strength cementitious composite also yields optimal properties. Uniform distribution is critical for the uniform distribution of internal forces [47]. Uneven distribution can create localized zones of increased stress concentration. Empty plastic bottles can also be used to create voids in the composite. The article is purely experimental in nature, and the authors do not provide the stress–strain state analysis of the structure near stress concentrators [48]. Mixing quality, the homogeneity of the distribution of spheres in the mixture and, consequently, the predictability of their effect on the properties of the material depends on it [49,50,51].

Under significant loads and deformations, it is necessary to consider the nonlinear behavior of the material: the dependence of the elastic modulus on the degree of deformation, and the occurrence and development of cracks [52,53]. This often requires the use of specialized software packages with appropriate solvers. Developers also face problems related to fire exposure and loads typical of marine and oceanic conditions [54,55,56,57].

This review allowed us to identify current areas for improving the design of the Bubble Deck Slab System based on the use of lightweight foam concrete with the inclusion of polypropylene spheres and to identify gaps in existing research. Issues related to the analysis of the structure’s behavior under non-uniform thermal fields, the characteristics of the stress–strain state (SSS) of the structure with hollow spheres during bending, and the influence of the geometry and material of the spheres and matrix on the durability and strain characteristics of the Bubble Deck Slab System remain unclear. Modeling the flexural behavior of structural foam concrete containing hollow spherical polypropylene fillers under thermal and mechanical loads presents a number of challenges and limitations. The hollow polypropylene spheres are randomly distributed within the foam concrete matrix, creating a heterogeneous structure. Under mechanical loading, this composite exhibits anisotropic properties. The failure of such a heterogeneous composite involves crack propagation, a process that is difficult to predict, particularly at the moment of failure. The aim of this study is to analyze the thermophysical properties of lightweight foam concrete incorporating hollow polymer spheres under the conditions of the Far North, to evaluate its flexural strength, and to investigate the influence of various polymer materials on the composite’s performance characteristics. The novelty of this work lies in the study of the flexural behavior of a composite comprising a concrete matrix with hollow polymer spheres under the combined action of thermal and external mechanical loads. While compressive behavior has been investigated previously, flexural properties under simultaneous thermal exposure have not been studied. A key feature of the study is the development of a numerical model in which the porous matrix incorporates polymer spheres; each sphere is modeled as a polymer shell possessing deformation stiffness and acting as a lightweight aggregate.

## 2. Materials and Methods

### 2.1. General Methodology of the Research

The general research process is based on the methodology of the authors’ previous studies [6,11,20]. The research process includes experimental laboratory tests, numerical analysis, and interpretation of the results. In this paper, experimental laboratory studies refer to a set of characteristics determined physically in the laboratory and through modeling using specialized software. The physical and mechanical properties of structural foam concrete with hollow polypropylene spherical fillers, such as density, compressive strength, and flexural strength, were determined through laboratory physical experiments. Thermal conductivity was determined experimentally only for the control composition of structural foam concrete.

Tests were conducted in an experimental lab, creating foam concrete samples. These samples included a standard mix, along with mixes comprising polypropylene spheres of these sizes: 10 mm, 12 mm, 15 mm, 19.05 mm, and 20 mm. To test density, compressive strength, flexural strength, and thermal conductivity, six series of specimens were prepared. Article [11] provides a detailed analysis of compressive strength and stress–strain behavior. This study focuses on the bending behavior of foam concrete containing polymer spheres of different sizes, even in a non-uniform thermal environment.

The finite element method (FEM) simulations in ANSYS (version 2022 R1) involved multiple steps. Initially, the two-phase solid model for foam concrete included air voids. This model served as the foundation for the next phase, which involved creating a concrete matrix for lightweight concrete using polypropylene spheres of the specified diameters. A comparison between experimental data and numerical simulations served to verify the model.

The third step suggested analyzing different polymers: polyamide 66 and polyethylene were substituted for polypropylene, and their behaviors were compared. At this stage, a comparative analysis of the effectiveness of mixtures with polymer spheres of various diameters was conducted.

### 2.2. Materials

**Figure 1:**
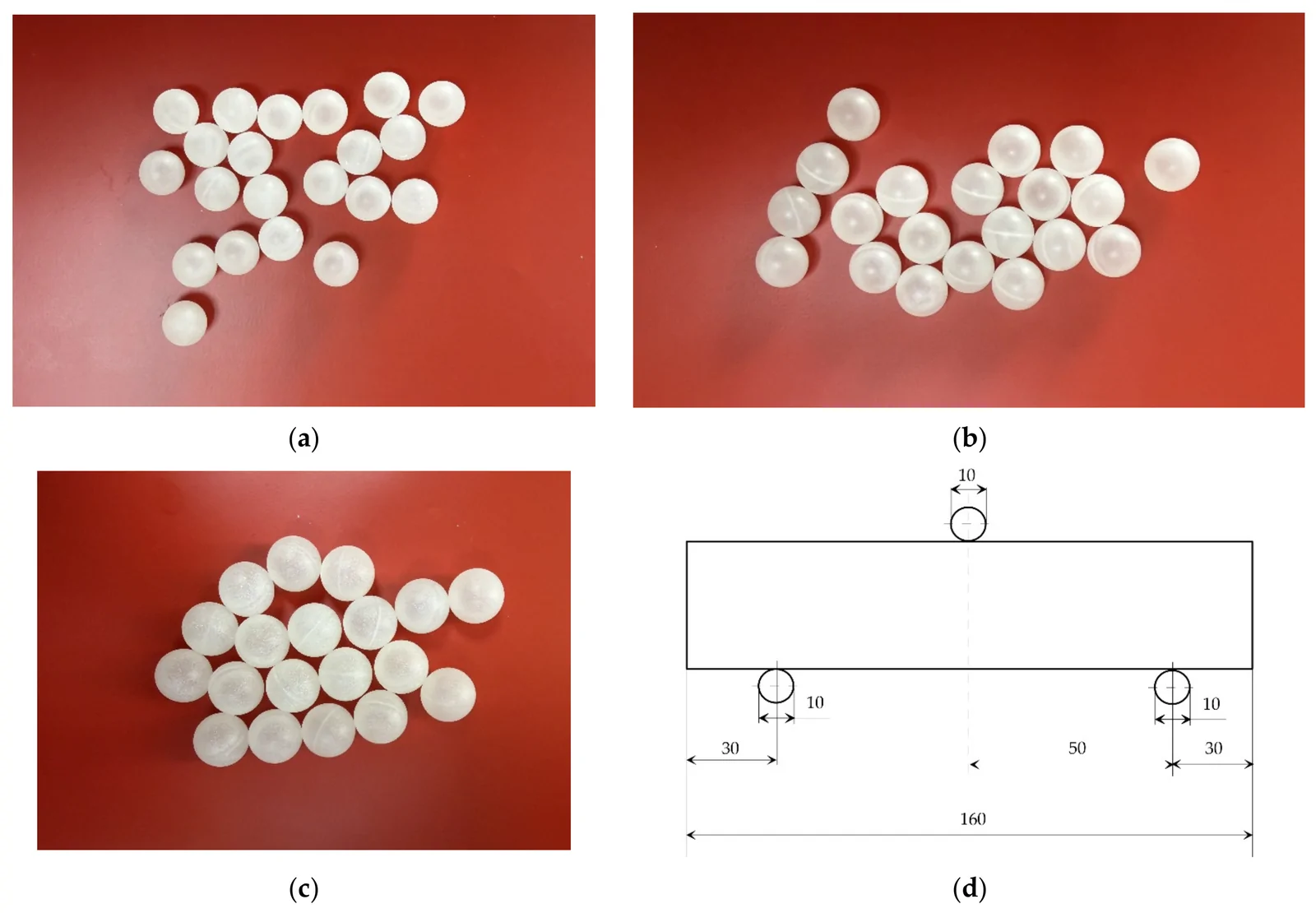
Polypropylene spheres and the loading scheme: (**a**) 10 mm; (**b**) 12 mm; (**c**) 15 mm; and (**d**) loading scheme for the determination of flexural strength.

To create the experimental structural foam concrete samples with hollow polypropylene spherical fillers, the following raw materials were utilized. Portland cement CEM I 42.5 N (PC) (CEMROS, Stary Oskol, Russia) properties: normal density—28%; setting time—200 min start, 280 min end; compressive strength after 28 days—51.3 MPa; flexural strength after 28 days—7.9 MPa. Quartz sand (QS) (Nedra, Samara, Russia) properties: bulk density—1346 kg/m^3^; fineness modulus—1.59. Foaming agent Rospena (R) (Rospena, Moscow, Russia) properties: composition—25% proteins, 4% mineral salts; density—1.1 g/cm^3^; Stability: 0.7–3.5 h. Polypropylene sphere (PPS) (Taian Xinxin Import & Export Co., Ltd., Taian, China) properties: base material—polypropylene; sphere diameters: 10, 12, 15, 19, and 20 mm; sphere wall thickness: 1 mm. Polypropylene spheres were used in the experiment, as well as for model calibration and verification. Simulation of the behavior of polyethylene and polyamide spheres was performed solely numerically to study the influence of the properties of other polymeric materials under similar stress–strain conditions. An image of the polypropylene spheres is shown in Figure 1.

Experimental structural foam concrete samples incorporating hollow spherical polypropylene fillers were created using the foam concrete mixture detailed in Table 1.

A total of six experimental compositions were produced with the following volume ratios of foam concrete mixture to polypropylene spheres (Table 2). The volume fraction of spheres was calculated based on the outer diameter of the spheres. In thermal and strength calculations, the sphere was considered as a shell with a thickness of 1 mm. Number of spheres in one sample: 10 mm—73 pcs.; 12 mm—42 pcs.; 15 mm—21 pcs.; 19 mm—11 pcs.; and 20 mm—9 pcs.

Several steps were involved in creating the experimental structural foam concrete samples with hollow polypropylene spherical fillers. Stage 1: Follow the recipe to prepare and dose the raw materials. Stage 2: Preparation of the foam concrete mixture. PC and QS were mixed dry, then mixing water and foaming additive R were added. First, Portland cement was loaded into the foam concrete mixer, followed by sand and water. The components were mixed for 2 min at 600 rpm in a 50 L turbulent laboratory foam concrete mixer CA 400/500 (DSTU, Rostov-on-Don, Russia) using a single-stage technology without separate foam preparation. A foaming agent was added to the resulting mixture, and the mixture was mixed at 600 rpm for 4 min. Stage 3: The finished foam concrete mixture was mixed with PS for 60 s. The resulting composition was then loaded into 40 × 40 × 160 mm metal prism molds and compacted by tapping the molds on a metal table 15 times. At this stage, the uniform distribution of the polypropylene spheres in the samples was visually checked. Stage 4: After 24 h, the composite samples were removed from the molds and stored in an air-dry condition at a temperature of 20 ± 2 °C and a relative humidity of 50 ± 10%. Each composition had 3 samples (batch size), making for a total of 18 experimental prism samples.

### 2.3. Experimental Methods

The properties of structural foam concrete with hollow polypropylene spherical fillers were evaluated under the requirements of standardized methods for the following characteristics: density; flexural strength; compressive strength; and thermal conductivity coefficient [58,59,60].

1. Using prism-shaped specimens, the composite density was measured after 28 days of exposure to air-dry condition at 20 ± 2 °C and 50 ± 10% relative humidity. The specimens’ dimensions were measured before they were weighed. Formula (1) was employed to calculate the density *ρ* of each specimen, allowing for an error margin of up to 1 kg/m^3^. Density is found by averaging the results from three tests.
  (1) $$\rho =\frac{m}{V}\times 1000$$
  Here *m* is the specimen mass (g); *V* is the specimen volume (cm^3^).
2. An S205 testing machine (Matest, Treviolo, Italy) was utilized to evaluate the compressive and flexural strength of the composites, employing the identical specimens from the density tests. Initially, the specimens were attached to the support parts of a specialized flexural strength tester. Next, the tester and specimen were placed in the testing machine, and a force was applied at a rate of 50 ± 10 N/s. After the flexural tests, the beam samples were positioned with their ends extending 10 mm past the compression plates. A loading rate of 2400 ± 200 N/s was used for compressive strength determination. Formulas 2 and 3 were used to determine the flexural and compressive strength of the specimens, with results accurate to 0.1 MPa. The flexural strength is determined by averaging three sample test outcomes, and the compressive strength is the average of six half-sample outcomes.
  (2) $$R=\frac{F}{A}$$
  Here *F* is the breaking load (N); *A* = 2500 (mm^2^).
  (3) $$R_{tb}=\delta \frac{Fl}{ab^{2}}$$
  Here *a*, *b*, and *l* are the width, height of the cross-section of the prism, and the distance between the supports (mm).

**Figure 2:**
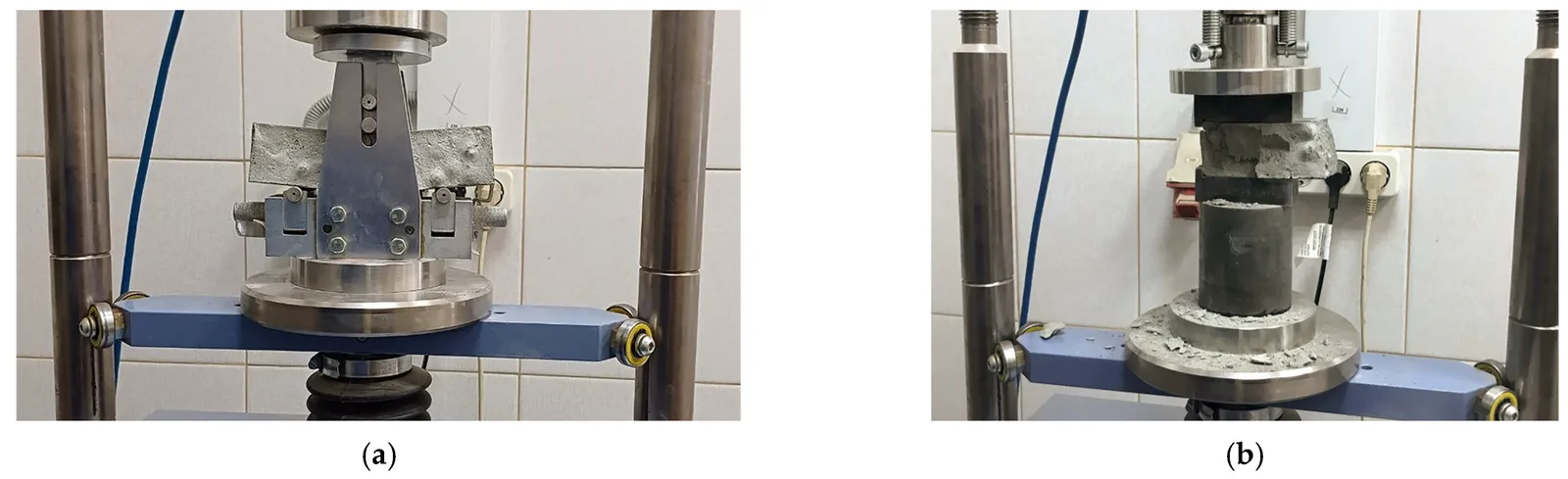
Experimental setup with structural foam concrete specimens containing polypropylene spheres: (**a**) determination of flexural strength; (**b**) determination of compressive strength.

The experimental setup for testing specimens of structural foam concrete containing polypropylene spheres is shown in Figure 2.

3. The thermal conductivity coefficient of structural foam concrete of the control composition was measured using an ITP-MG4 device (SKB Stroypribor, Russia) in accordance with the requirements of the standardized method on specimens measuring 100 × 100 × 20 mm [60]. Initially, the specimens were inspected for deviations of the front faces from parallelism. This deviation did not exceed ±0.5 mm. If deviations exceeded the norm, the specimens were ground. Then, using a caliper, the specimen thickness was measured with an error of no more than ±0.1 mm at four corners at a distance of (50 ± 5) mm from the corner apex and the center of each side. Before testing, all composite specimens were dried in a drying oven at a temperature of 50 ± 5 °C until constant weight was reached. The dried specimens were placed in the device, and the measurement process was carried out. The thermal conductivity coefficient of the control composition foam concrete was determined as the arithmetic mean of the test results of five samples. The device’s heater temperature control range is 25–60 °C. The permissible relative error limit for thermal conductivity measurements under steady-state conditions is ±5.0%. Before measuring thermal conductivity, the device underwent all necessary calibration and adjustment procedures.

## 3. Results and Discussion

### 3.1. Results of Determining the Properties of Structural Foam Concrete with Hollow Polypropylene Spherical Fillers

The results of determining the density, compressive strength and flexural strength of experimental samples of structural foam concrete with hollow polypropylene spherical fillers of different diameters are presented in Table 3.

As can be seen from Table 3, the density of structural foam concrete decreased with the inclusion of various types of polypropylene spherical fillers. The largest density reduction of 7% was recorded for the 10 PS composition. Compressive strength also decreased with the inclusion of polypropylene spheres [11]. The maximum reduction in compressive strength of 23.9% was recorded for the 12 PS composition. The maximum reduction in flexural strength of 36% was recorded for the 20 PS composition. The standard deviations (*S*_m_) and variation coefficients (*V*_m_) for each experimental composition are presented in Table 4.

Table 5 presents the results of the statistical analysis of variance of the experimental data.

The results of density measurements for structural foam concrete samples containing polypropylene spheres of various diameters show statistically significant differences: F(2; 10) = 22.88, *p* < 0.001 (below the 0.05 significance level). Compressive strength results also differ significantly: F(5; 25) = 23.61, *p* < 0.001 (below the 0.05 significance level). Flexural strength results show statistically significant differences: F(2; 10) = 44.13, *p* < 0.001 (below the 0.05 significance level). Thus, the analysis of variance confirms that incorporating polypropylene spheres of various diameters into the foam concrete matrix significantly affects the composite’s performance properties.

**Figure 3:**
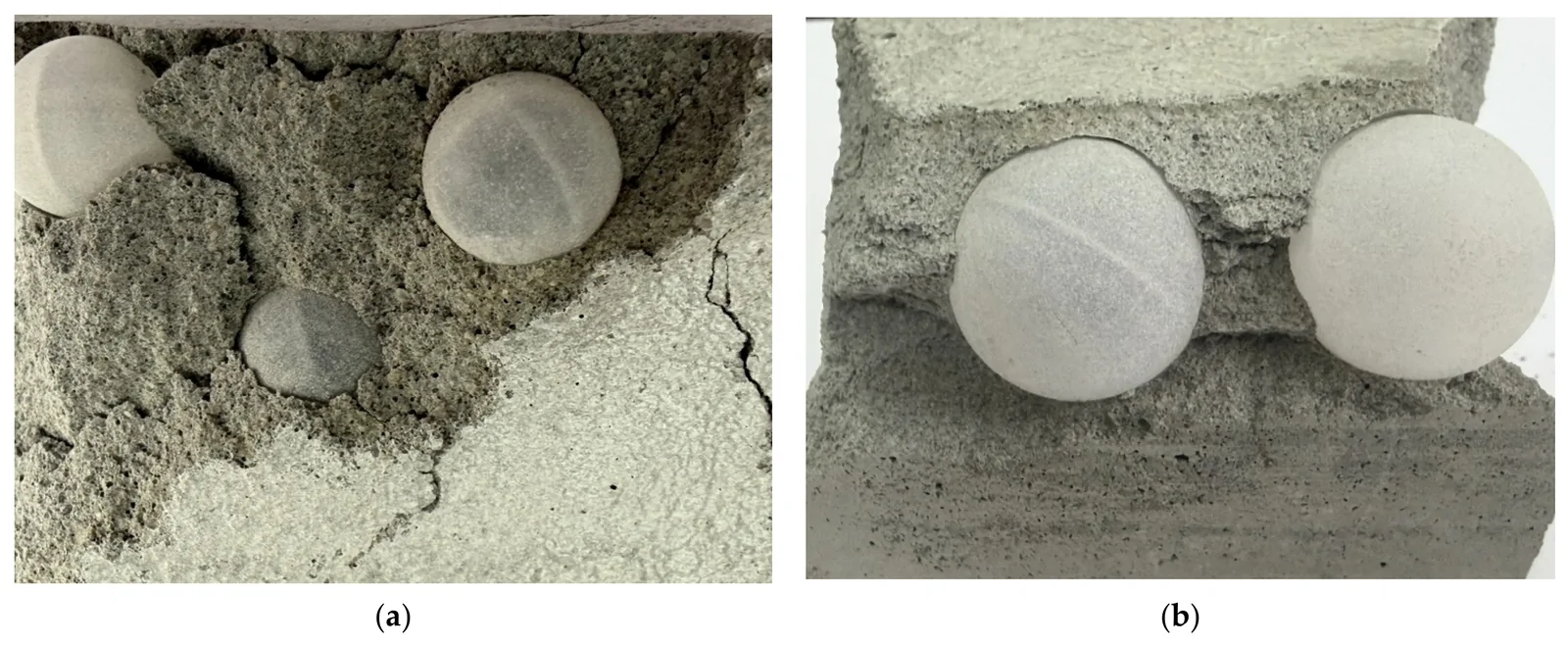
Photographs of the fracture of a destroyed specimen of structural foam concrete with polypropylene spheres: (**a**) fracture 1; (**b**) fracture 2.

The appearance of fractures of destroyed samples of structural foam concrete with polypropylene spheres is shown in Figure 3.

The structure of the foam concrete matrix on the fracture surface is homogeneous and rough, with a fairly uniform distribution of pores. Cracks and chips are visible. The contact zone of the foam concrete matrix conforms to the spherical shape of the polypropylene sphere. Cracks propagate in the foam concrete matrix in the areas between the hollow polypropylene spheres. Small gaps formed during the fracture process are visible at the interface between the foam concrete matrix and the polypropylene sphere. The macrostructure images shown in Figure 3 illustrate the structural changes and the nature of the defects that appear in the composite following mechanical strength testing. Gaps form at the “foam concrete matrix–polypropylene sphere” interfaces within the composite under the influence of loads. These zones represent the weakest points, and it is precisely at these interfaces that the composite’s structure begins to fail when subjected to mechanical loads.

### 3.2. Numerical Modeling of the Thermomechanical Properties of Foamed Concrete with Hollow Polypropylene Spherical Fillers

**Figure 4:**
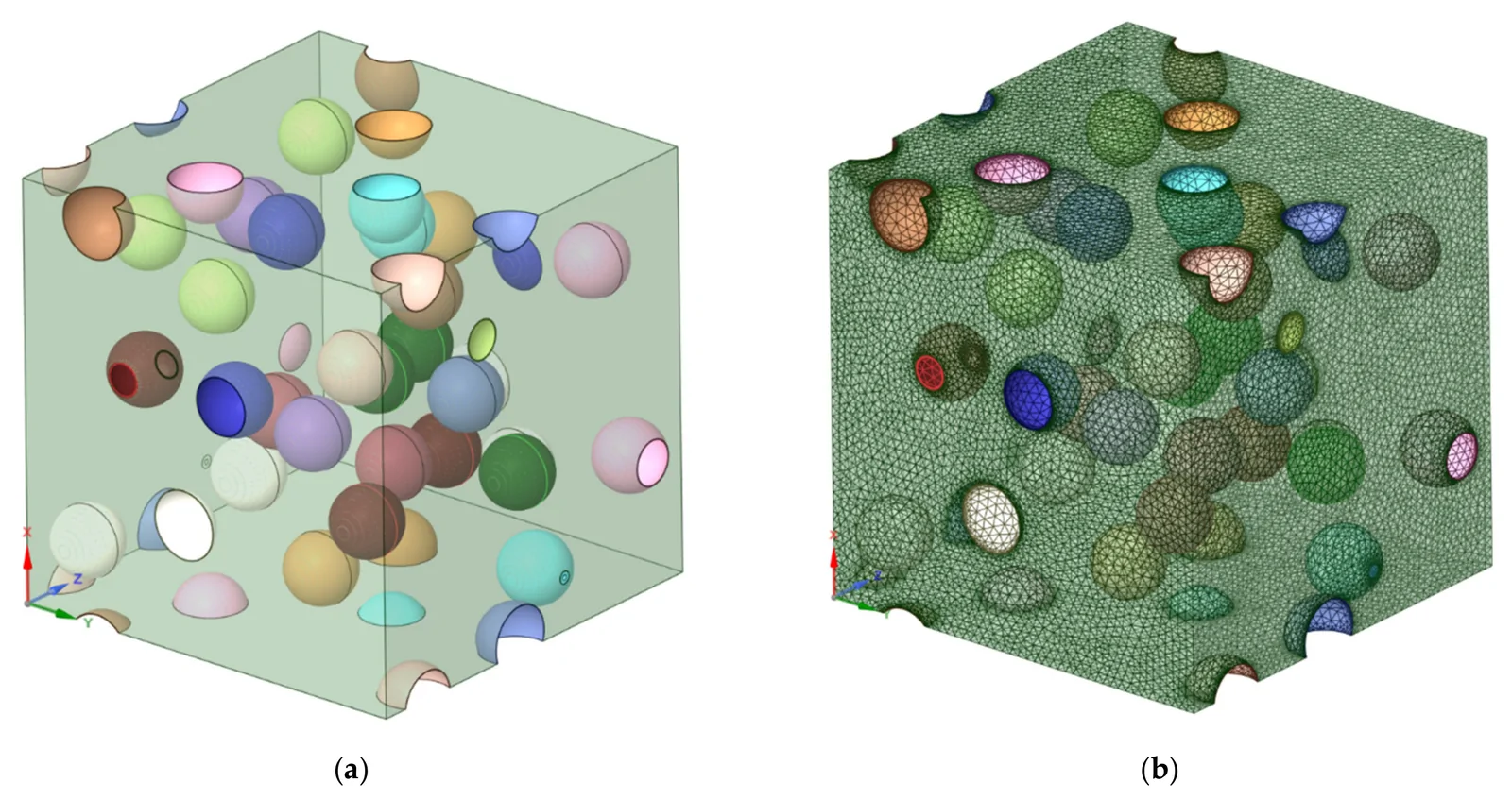
RVE model for determining the thermophysical properties of foam concrete with hollow polypropylene spherical fillers: (**a**) geometric model; (**b**) FEM model.

For subsequent calculations of the thermal fields and stress–strain state of a composite made of foamed concrete with hollow polypropylene spherical fillers, it is necessary to know the key parameters included in the thermodynamic equations. For this purpose, representative volumes of the composite with polymer spheres measuring 10 mm, 12 mm, 15 mm, 19 mm, and 20 mm were modeled in the Material Designer module, corresponding to experimental studies. Figure 4 illustrates the Representative Volume Element (RVE) model employed for calculating the composite’s thermophysical properties. Rigid bonding conditions are specified at the interface in the model.

The properties of the materials used for the calculation are presented in Table 6.

The density of the composite with polymer spheres, obtained in Material Designer, is presented in Table 7.

**Figure 5:**
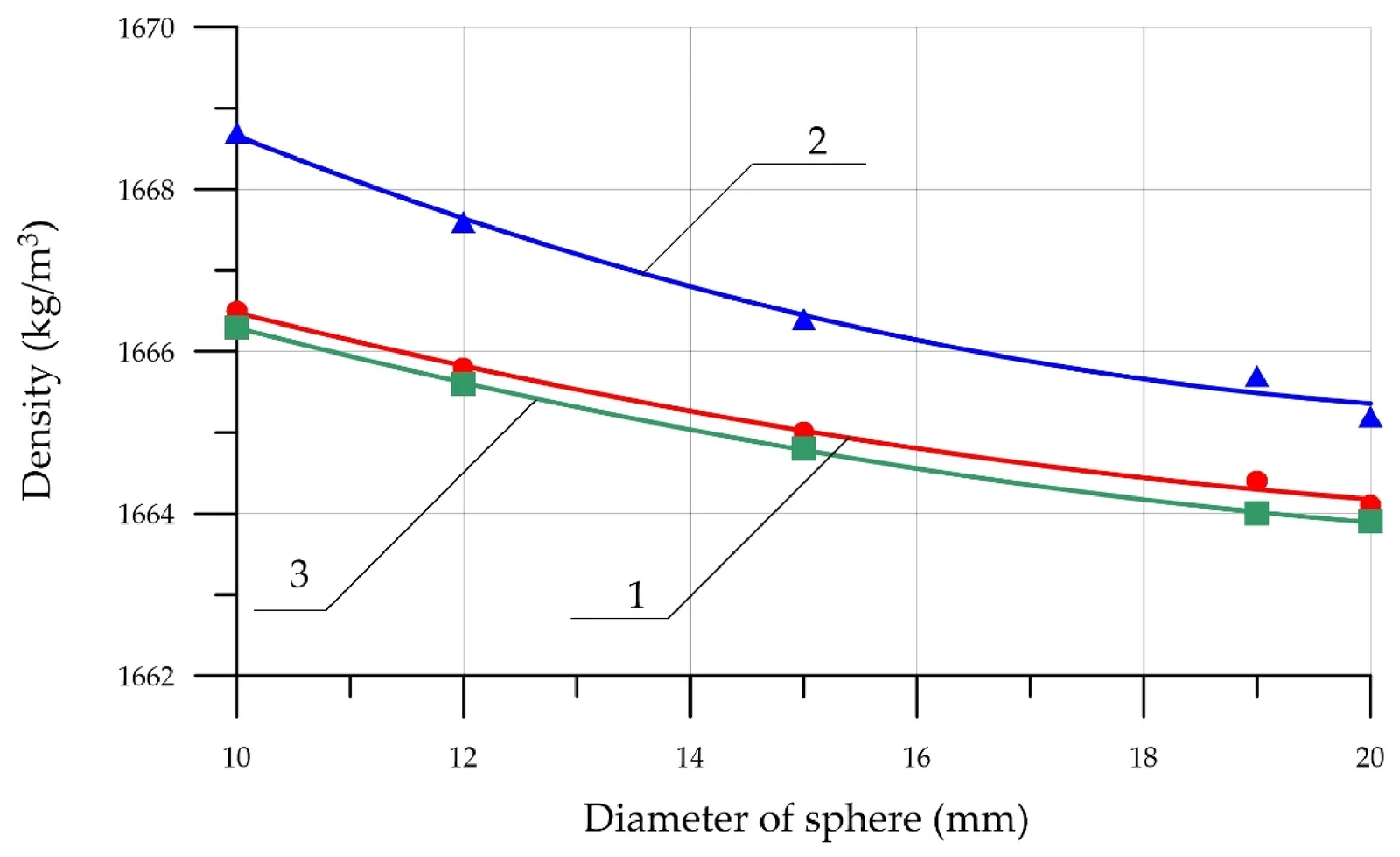
Density of a lightweight foam concrete composite as a function of the diameter of the polymer spheres: (1) polyethylene; (2) polyamide; (3) polypropylene.

Figure 5 illustrates the change in density of the composite with hollow polymer spheres.

**Figure 6:**
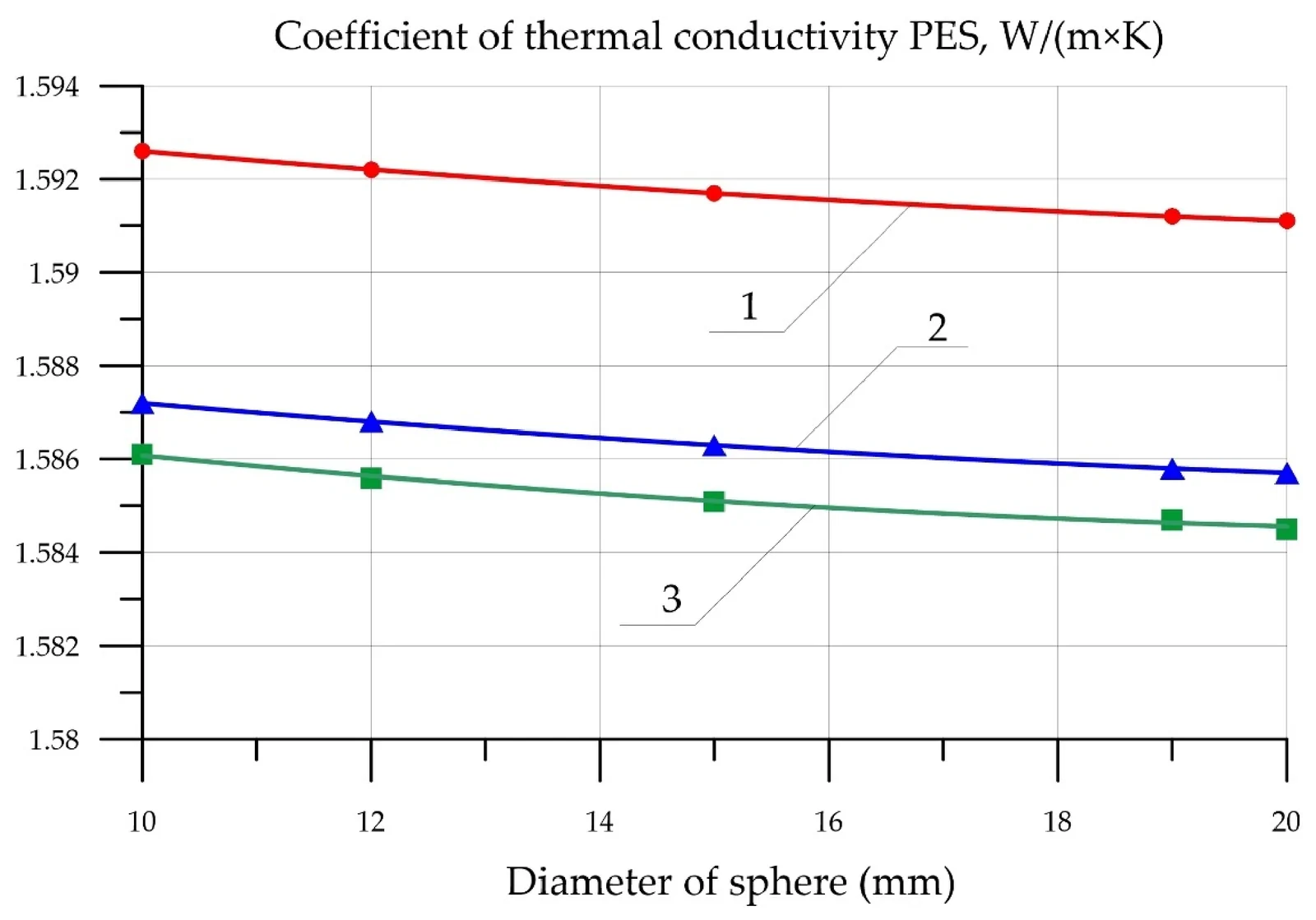
Dependence of the thermal conductivity coefficients of a composite with hollow polyethylene spheres: (1) *K_xx_*; (2) *K_yy_*; and (3) *K_zz_*.

**Figure 7:**
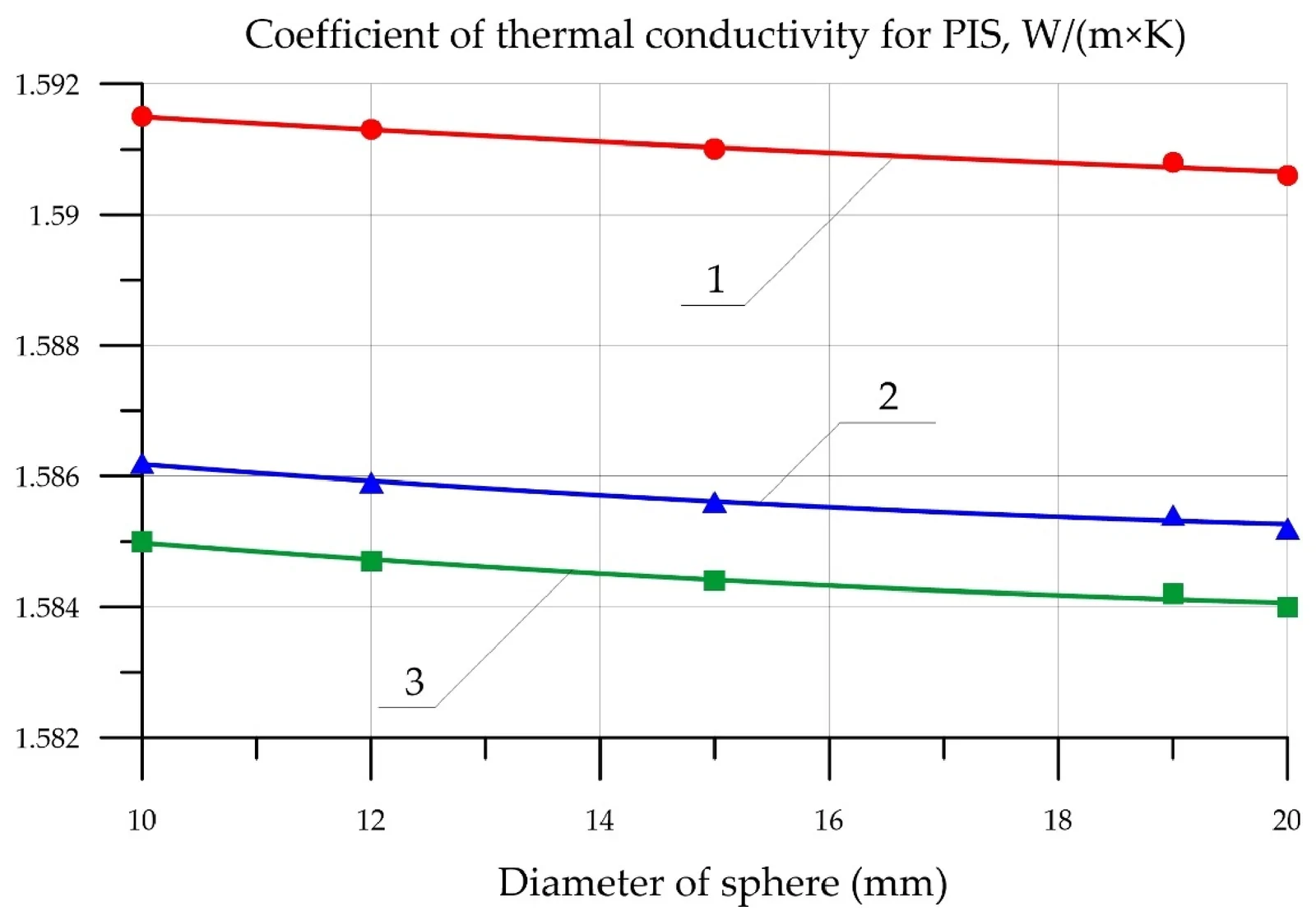
Dependence of the thermal conductivity coefficients of a composite with hollow polyamide spheres: (1) *K_xx_*; (2) *K_yy_*; and (3) *K_zz_*.

**Figure 8:**
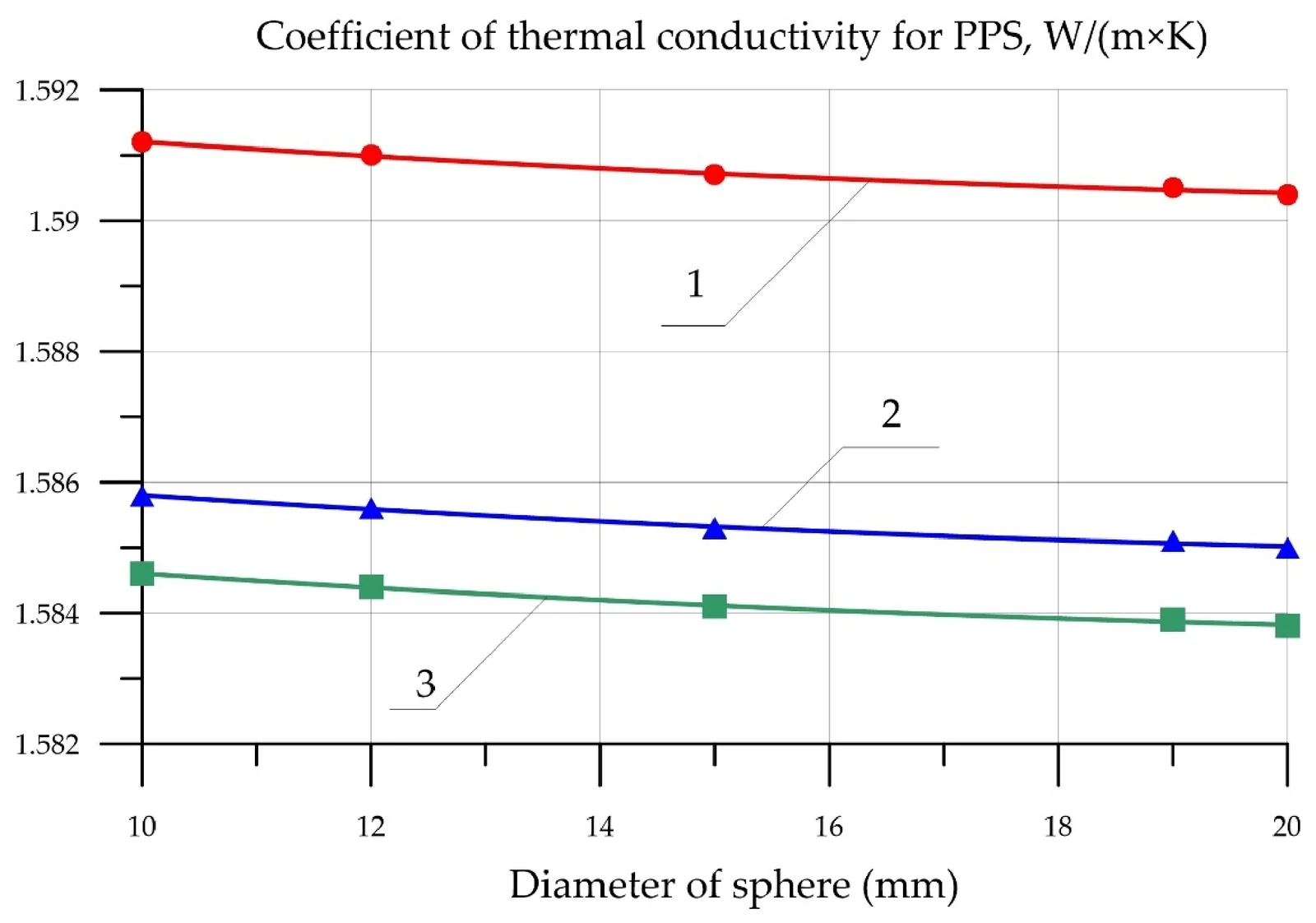
Thermal conductivity coefficients of a composite with hollow polypropylene spheres: (1) *K_xx_*; (2) *K_yy_*; and (3) *K_zz_*.

Figure 6, Figure 7 and Figure 8 illustrate the transformation in thermal conductivity coefficients of a composite with hollow polyethylene, polyamide, and polypropylene spheres.

Figure 6, Figure 7 and Figure 8 show that the introduction of hollow spheres (polyethylene, polyamide, or polypropylene) into the foam concrete matrix reduces the overall thermal conductivity of the composite compared to the original matrix. This occurs because the sphere walls and the air filling them have significantly lower thermal conductivity than the polymer. It is also evident that the dependence on sphere diameter is nonlinear. The change in thermal conductivity is not always directly proportional to the increase in the volumetric content of spheres. Other factors also influence the result: the size and shape of the spheres, their distribution in the matrix, and the quality of interfacial interaction (the adhesion of the polymer to the sphere wall).

**Figure 9:**
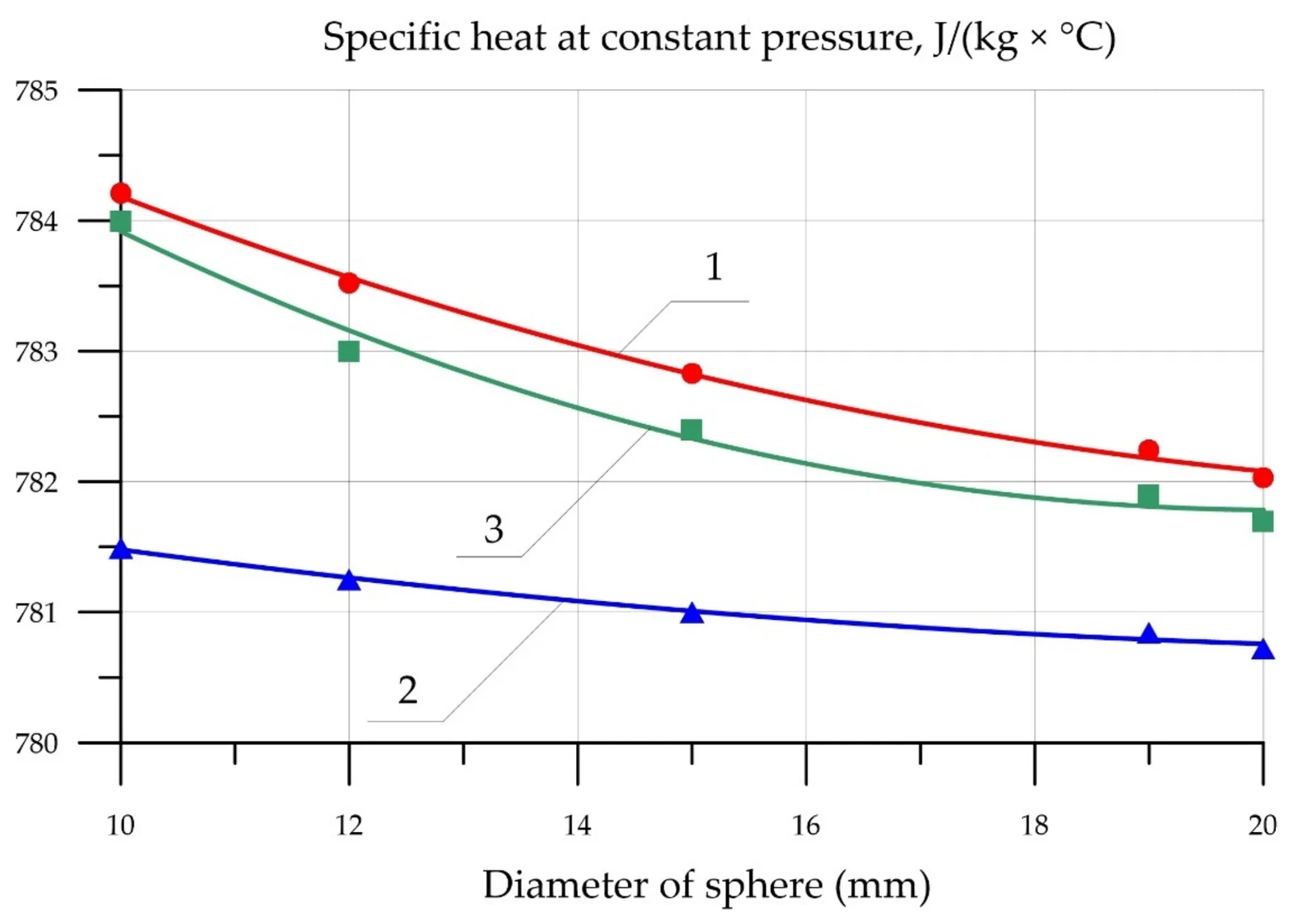
Specific heat capacity at constant pressure for a composite with different polymer sphere materials: (1) polyethylene; (2) polyamide; and (3) polypropylene.

The specific heat capacity at constant pressure for a composite with different polymer sphere materials is shown in Figure 9. Specific heat capacity is defined as the quantity of heat energy necessary to elevate the temperature of one unit of mass of a substance by one kelvin (or degree Celsius) under constant pressure. Specific heat capacity at constant pressure is used in subsequent calculations to determine the temperature field and the stress–strain state of the composite caused by this temperature field.

Figure 9 shows the specific heat capacity at constant pressure (*c*_p_) for a composite with polymer spheres. The result is influenced by several factors, primarily the properties of the polymer spheres themselves. Different materials have different specific heat capacities. For example, spheres made of polyethylene or polypropylene will behave differently in a composite. Polymers with a more complex molecular structure or with additional functional groups (such as some plasticizers or stabilizers) contribute differently to the overall heat capacity than simpler structures.

Knowing these relationships, further calculations can be used to select the composite composition for a specific task. For example, if a material with low heat capacity for rapid heating/cooling is needed, a cement matrix with a low *c_p_* and/or a large number of small spheres can be selected. However, if increased heat capacity is required (for example, for a thermos), it is advisable to select a matrix with high intermolecular bond energy or a certain filler content. In our case, to determine the uneven temperature field caused by temperature differences, as well as to determine the stress–strain state of the composite, it is necessary to know all of the thermophysical properties.

### 3.3. Features and Verification of the FEM Model for the Flexural Behavior of Structural Foam Concrete with Hollow Polymer Spherical Fillers

Finite element modeling (FEM) of the flexural behavior of structural foam concrete with hollow polymer spherical fillers (polypropylene spheres) is a complex task that requires consideration of the composite structure and careful model verification. When constructing an FEM model for a composite material, several key aspects must be examined.

A significant factor is accounting for the porosity of the cement matrix. To implement the model, the results of the thermophysical characteristics simulation obtained in Stage 1 of Material Designer are used as input data.

Hollow spherical filler elements, randomly distributed in the cement matrix, create a complicated, heterogeneous structure in the composite. This is realized in the model by introducing effective elastic moduli of the porous matrix, which take into account the deformation properties of the cement paste and pores. The spheres’ elastic and nonlinear properties, as well as the contact conditions between them, are defined in the model as separate initial characteristics. It is necessary to correctly specify the spatial distribution of the fillers in the volume, their size, and density, as these parameters directly affect the overall stiffness and flexural strength of the material.

Tetragonal elements with intermediate nodes are used for numerical modeling. For steady-state thermal modeling, SOLID291 is employed. This is a 3D, 10-node tetrahedral thermal finite element with a temperature degree of freedom. For the coupled strength-thermal analysis, SOLID187, a three-dimensional (3D), ten-node tetrahedral finite element, is used. A total of 25,244 elements and 15,794 nodes with a minimum element size of 3 mm are used in the calculation. To verify the mesh, preliminary calculations were performed using meshes of various resolutions. The error relative to the selected mesh (containing 25,244 elements) was as follows: 64.1% for 5426 elements, 33.4% for 10,341 elements, 5.1% for 20,581 elements, 0.5% for 25,101 elements, and 0.1% for 31,951 elements.

The behavior of foam concrete under bending is nonlinear: elastic-plastic deformations, crack development, and propagation are observed. In the FEM model, this requires the implementation of appropriate criteria dependencies in the form of the Menetrey–Willam model, which relate stress and strain in numerical integration [6,11]. The parameters of the Menetrey–Willam model were adjusted in accordance with recommendations and presented in Table 8 [11,20]. Material softening was represented using an exponential function and initiated once the plastic potential function reached its peak value.

The eccentricity is determined by the loading scheme (Figure 1d); viscosity is not taken into account in the Menetrey–Willam model. Specific conditions arise at interfaces (cement stone—air, filler particles—cement stone). Their correct specification is critical for accurate prediction of the stress–strain state.

The model reflects the geometry of the test specimen, a 40 × 40 × 160 mm beam, and a three-point bending loading pattern. Under real-world operating conditions, the material is subject to moisture and temperature changes. These factors must be taken into account in the model through appropriate boundary conditions and material laws.

Three types of polymeric materials were considered as spheres: polypropylene, polyamide, and polyethylene. For numerical modeling, the strength properties of the polymeric materials were specified using a bilinear model with isotropic hardening. The material properties are presented in Table 9.

**Figure 10:**
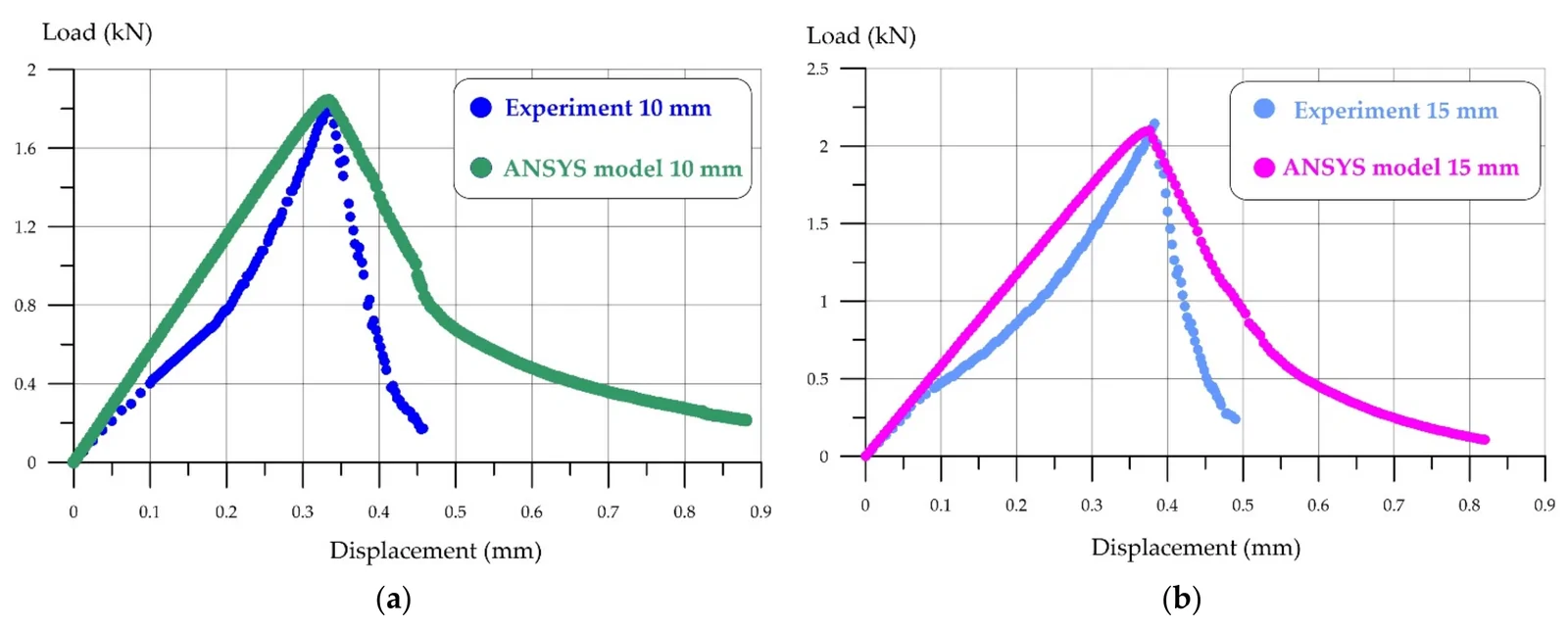
Comparison of experimental data and numerical simulation results for a composite with polypropylene spheres: (**a**) 10 mm diameter; (**b**) 15 mm diameter.

To verify the FEM model, a series of flexural tests were conducted on specimens with varying filler volume contents, sphere sizes, and curing conditions. Key parameters measured included the load–deflection curve, flexural strength, and crack propagation pattern. Nonlinear component models (cement aggregate and polypropylene spheres) were calibrated for FEM implementation using the experimental data. Figure 10 presents a comparison between experimental data and numerical simulation outcomes for a composite containing 10 mm and 15 mm diameter polypropylene spheres.

The model successfully captures the primary loading and unloading processes, as confirmed by comparing experimental data to the numerical simulation results shown in Figure 10, with respect to absolute strength and deformation values. The main discrepancy is evident in the region of the descending load–displacement curve. This is also noted in [6,11,20,27]. For brittle cementitious materials, such as this composite, failure under bending or tension occurs quite quickly, which is poorly accounted for by the Menetrey–Willam model. Table 10 shows the errors in modeling the peak load and the displacement corresponding to the peak load.

Nevertheless, the model adequately describes the loading stage and displays the strength and deformation characteristics, allowing its application in subsequent stages: stress–strain analysis under non-uniform temperature conditions and during bending of the composite with polymer spheres.

One limitation of the proposed model is that, due to symmetry, it represents only half of the sample. The spheres within it are described as randomly distributed. Random filler distribution is typically asymmetric, which leads to inaccuracies. Additionally, the model assumes conditions of perfect bonding at the phase interface. In actual samples, interfacial behavior involves slippage; however, the lack of sufficient research on this issue prevents the accurate modeling of interfacial bonding relationships.

### 3.4. Modeling the Stress–Strain State Under Non-Uniform Thermal Conditions

Analyzing the bending of a foam concrete slab in Arctic conditions is a complex engineering problem, where the material’s behavior is influenced by harsh climatic factors and the properties of the material itself. In Arctic conditions, the slab is subject to a range of adverse effects, including an uneven temperature field. Temperatures can be distributed unevenly not only across the slab’s thickness but also across its surface due to wind, shading by surrounding objects, and heat exchange with the soil or water.

Arctic conditions are subject to sharp temperature fluctuations. The surface can heat up during the day (for example, from the sun) and cool down sharply at night. This creates internal temperature gradients. Water, which may be contained in the pores of the foam concrete, expands when it freezes. This creates additional internal pressure and stresses that can damage the structure of the material.

Bending occurs because different parts of the slab heat or cool to different degrees. This creates internal forces: some layers of the slab tend to expand, while others tend to contract. Uneven heating and associated stresses cause complex deformation processes in the foam concrete due to an uneven stress–strain state. This causes the slab to become less rigid overall because of how material properties change with temperature, increasing the risk of cracking. In Arctic conditions, where the material is already weakened by freezing cycles, this greatly accelerates failure.

**Figure 11:**
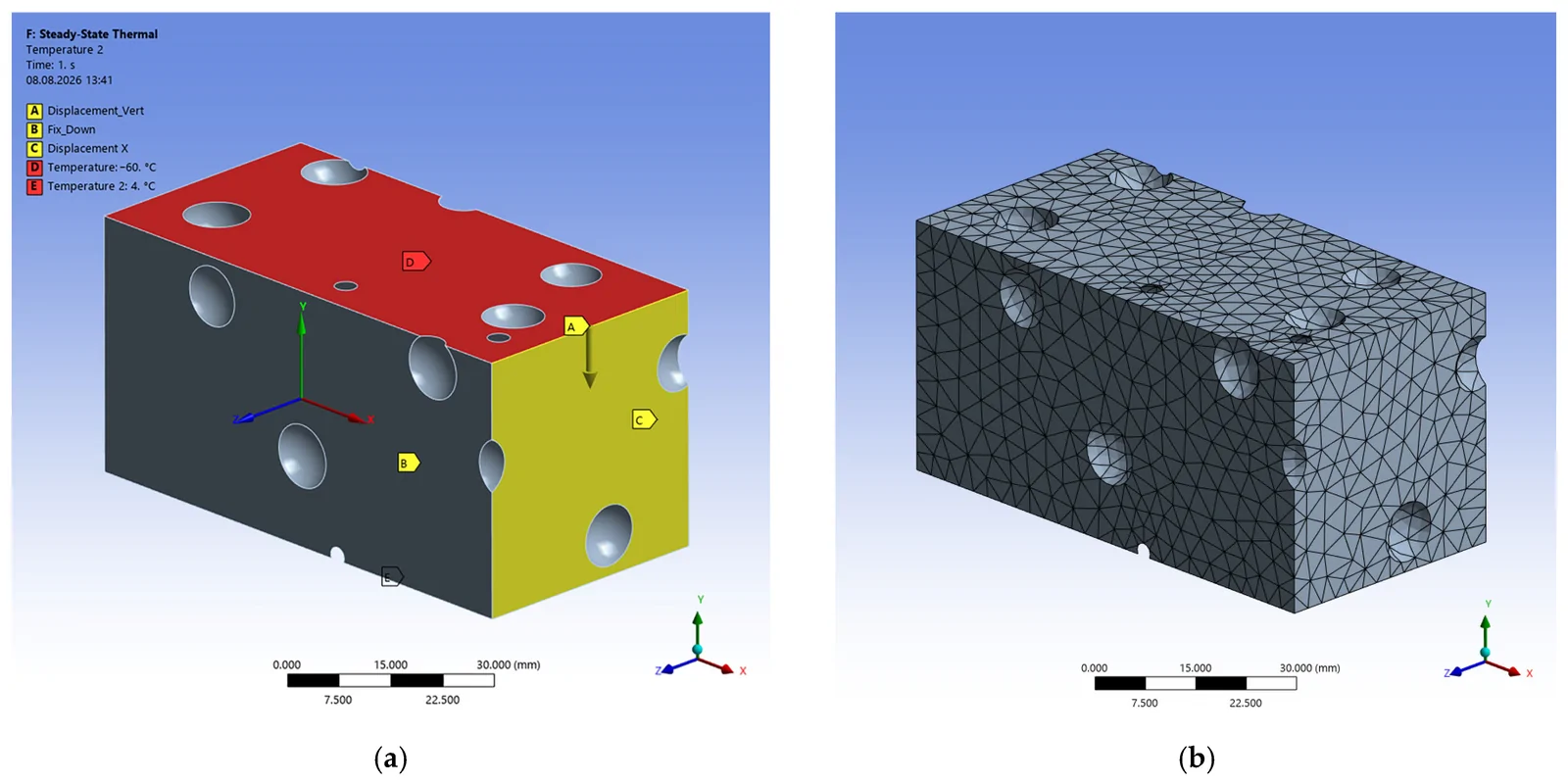
FEM model: (**a**) geometric model and boundary conditions; (**b**) finite element mesh.

Let us consider two problems concerning the stress–strain state of a slab made of high-strength foam concrete with polymer spheres. The body occupies a position in Cartesian space 0≤x≤l, 0≤y≤a, 0≤z≤b , where *a* = *b* = 40 mm and *l* = 160 mm. Due to the symmetry of the problem, we consider half of it (Figure 11a,b).

The problems were solved under the following boundary conditions.

***Problem 1.*** All nodes lying on the support line x=30 mm, 0≤y≤40 mm, z=0 are fixed in the Y direction, i.e., $U_{Y}$ = 0. Due to the symmetry of the problem, all nodes belonging to the plane x=80 mm, 0≤y≤40 mm, 0≤z≤40 mm (Figure 10a, highlighted in yellow) are fixed in the X direction, i.e., $U_{X}=0$. A temperature of −60 °C is applied to the upper plane. A temperature of +4 °C is applied to the lower plane 0≤x≤80 mm, y=0 mm, 0≤z≤40 mm.

***Problem 2*****.** In accordance with the regulatory document (EN 12390-7:2019 [58]), a displacement δ is applied along the line x=80 mm, y=40 mm, 0≤z≤40 mm to model bending. The temperature on the upper and lower planes is the same and equal to room temperature.

To analyze the temperature field in problem 1, the Fourier equation was solved

(4) $$\frac{\partial T}{\partial t}-K\;\Delta T=f\left(r,\;t\right)$$

Here *T* is the temperature, *t* is the time, **K** is the matrix of thermal conductivity coefficients, and **r** = (*x*,*y*,*z*) is the Cartesian coordinate space.

In matrix form, when discretizing space

(5) $$\left\{q\right\}=-\left[K\right]\left\{L\right\}T$$

Here $K=\left(\begin{array}{ccc} K_{XX} & 0 & 0\\ 0 & K_{YY} & 0\\ 0 & 0 & K_{ZZ} \end{array}\right)$ is a matrix of thermal conductivity coefficients;

$\left\{q\right\}$ is a heat flux vector; $\left\{L\right\}=\left\{\begin{array}{l} \frac{\partial }{\partial x}\\ \frac{\partial }{\partial y}\\ \frac{\partial }{\partial z} \end{array}\right\}$ is a vector-operator.

**Figure 12:**
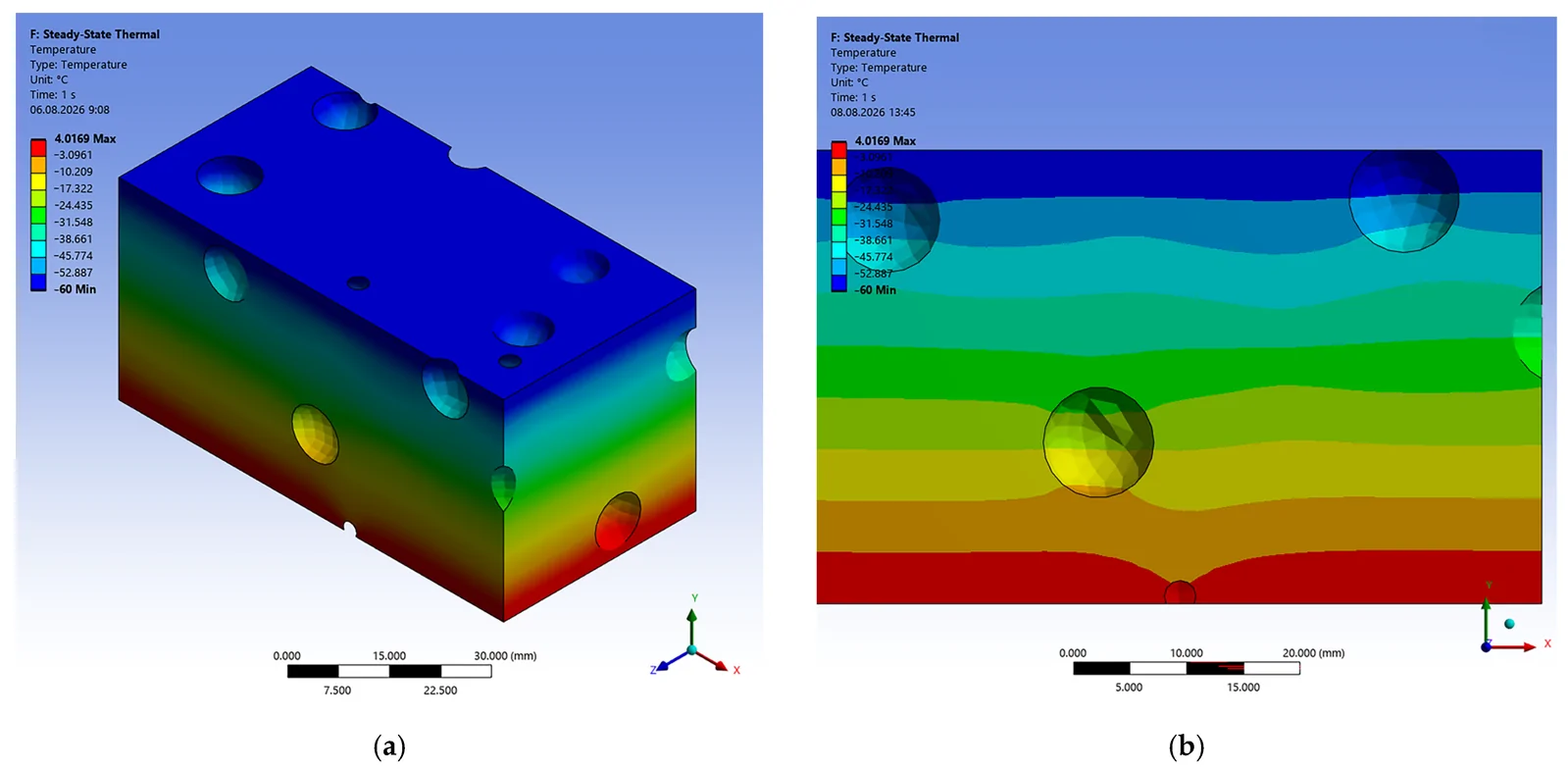
Temperature field in composite foam concrete with polymer spheres: (**a**) isometric view; (**b**) field non-uniformity near the polymer spheres.

The solution of Equation (5) in the form of a temperature field in a foam concrete sample is shown in Figure 12.

**Figure 13:**
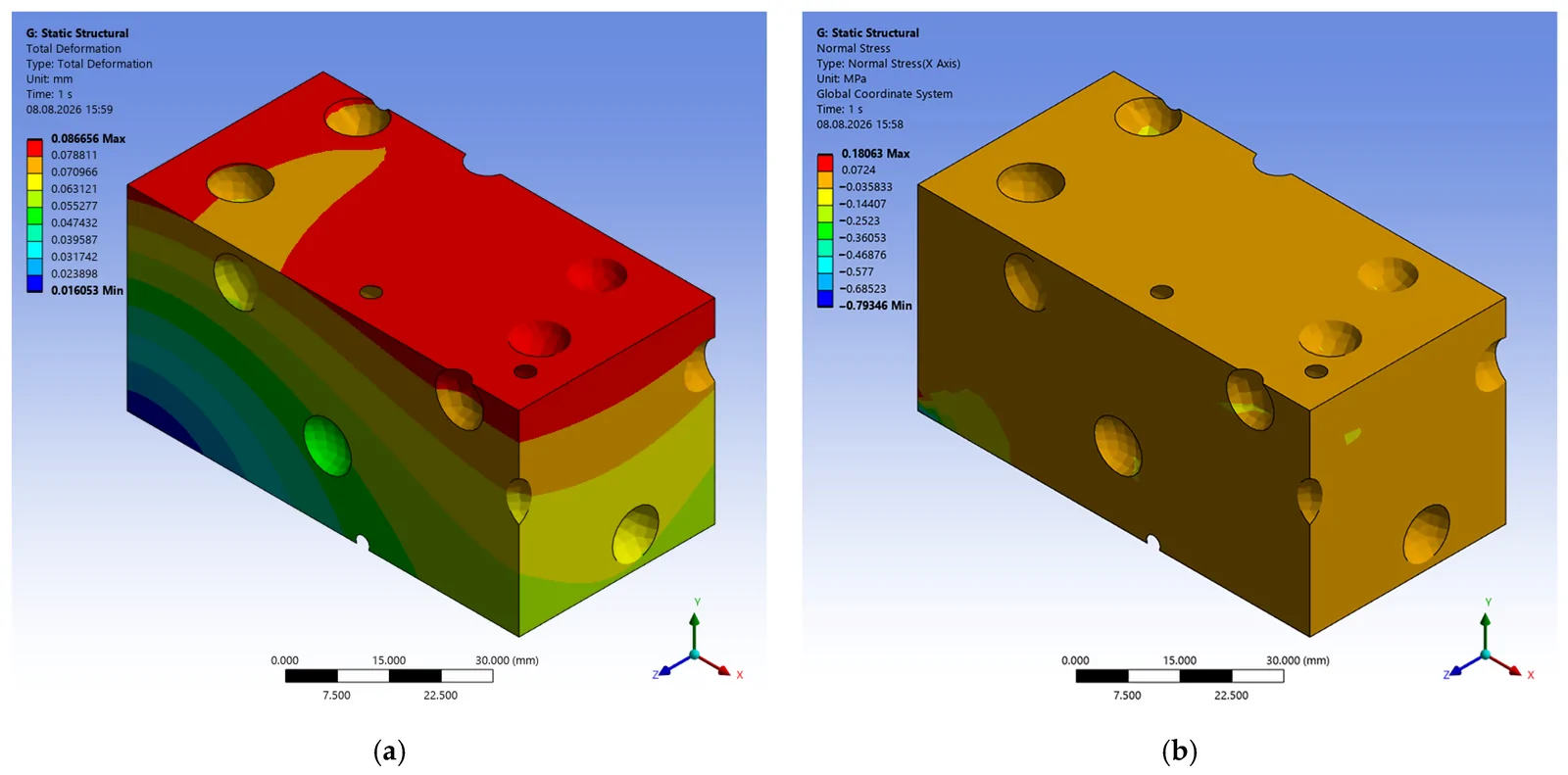
Stress–strain state of a composite beam with polymer spheres under non-uniform temperature influence: (**a**) total strain (mm); (**b**) stress *σ*_X_ (MPa).

The temperature field diagrams show that spatial non-uniformity occurs when the block cools unevenly. On the one hand, there is a large difference in temperature between the lower plane (presumably water) and the upper plane, which in the Far North can reach –60 °C. This is compounded by the non-uniformity caused by the presence of hollow spheres, whose thermophysical properties differ from those of the cement matrix. This non-uniformity creates a stress–strain state, which leads to the deformation of the entire structure. In the next step, the resulting temperature field was entered as input for a static calculation of the stress–strain state caused by the non-uniform temperature field (Figure 13).

The calculation results showed that the maximum equivalent von Mises stress was 3.3 MPa, indicating that the material operates in the elastic zone. The maximum displacement due to the temperature difference (Figure 13a) was 0.087 mm, and the tensile stress in the longitudinal direction (Figure 13b) was *σ*_X_ = 0.18 MPa.

### 3.5. Modeling the Behavior of Structural Foam Concrete with Hollow Polypropylene Spherical Fillers Under Bending

**Figure 14:**
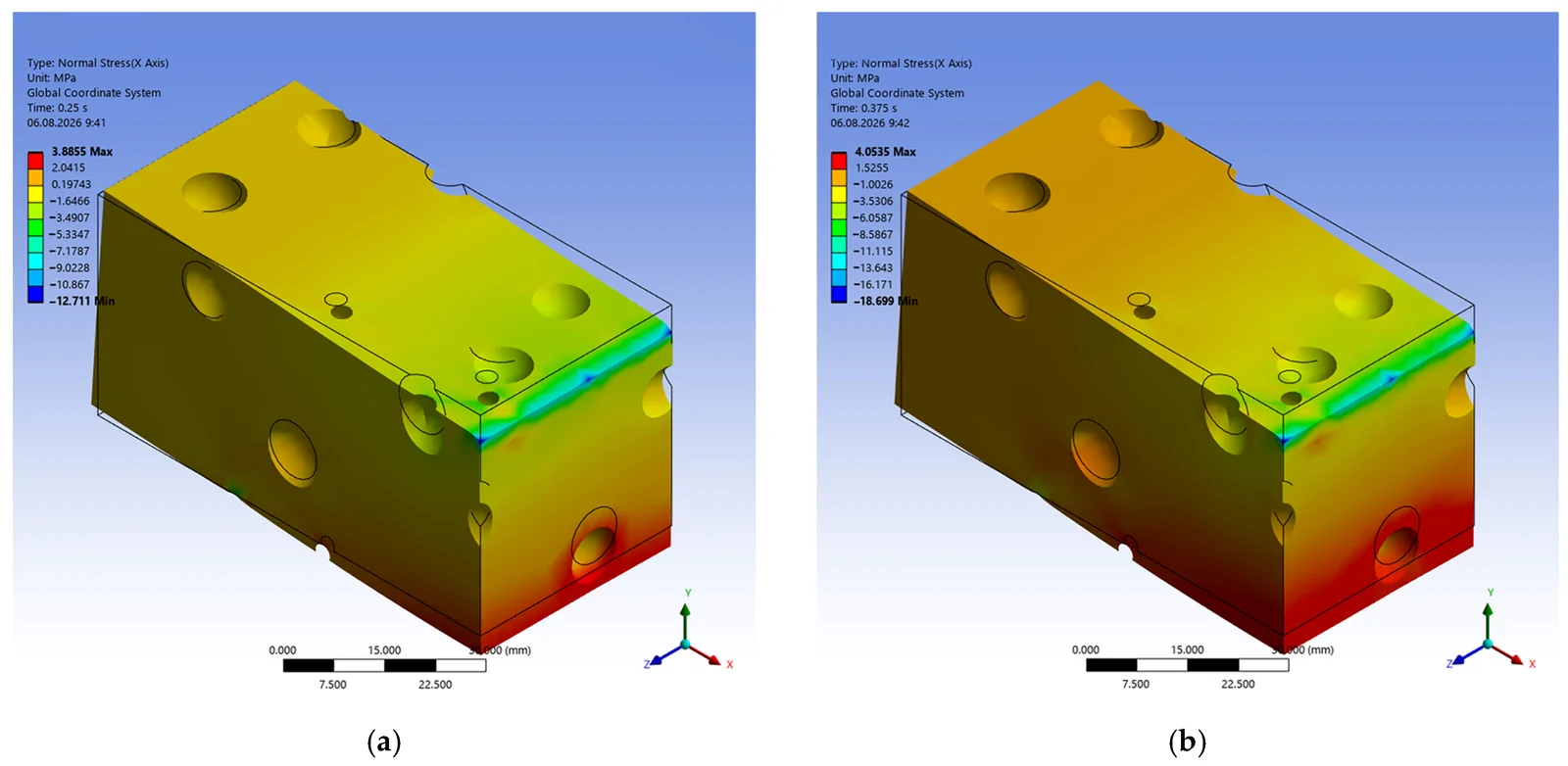

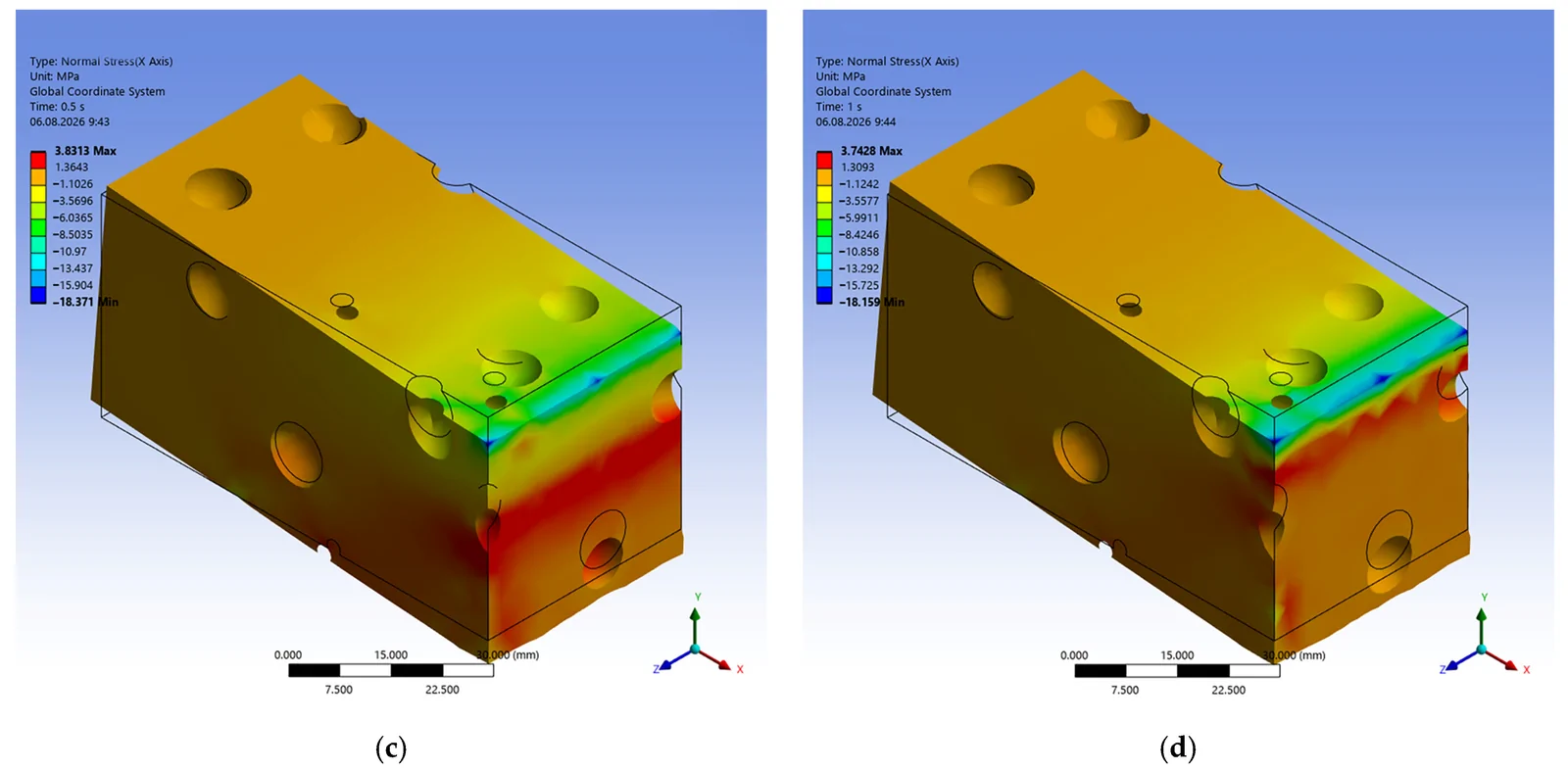
Stress fields *σ*_X_ during bending: (**a**) at 25% of the maximum load; (**b**) at 37.5% of the maximum load; (**c**) at 50% of the maximum load; and (**d**) at 100% of the maximum load.

Consider Problem 2, described in Section 3.3, without taking into account the thermal influence. Figure 14 shows the stresses in the longitudinal direction σ_X_.

Figure 14 shows the stress fields *σ*_X_ at various loading stages. The red zone corresponds to the maximum tensile stress. It can be seen that already at 37.5% of the maximum load, the tensile stresses reach 4 MPa and cracking begins. Figure 14c shows that the red zone shifts upward along the cross-section, indicating that the crack grows and reaches 1/3 of the cross-section. Figure 14d shows that almost the entire cross-section has already failed.

**Figure 15:**
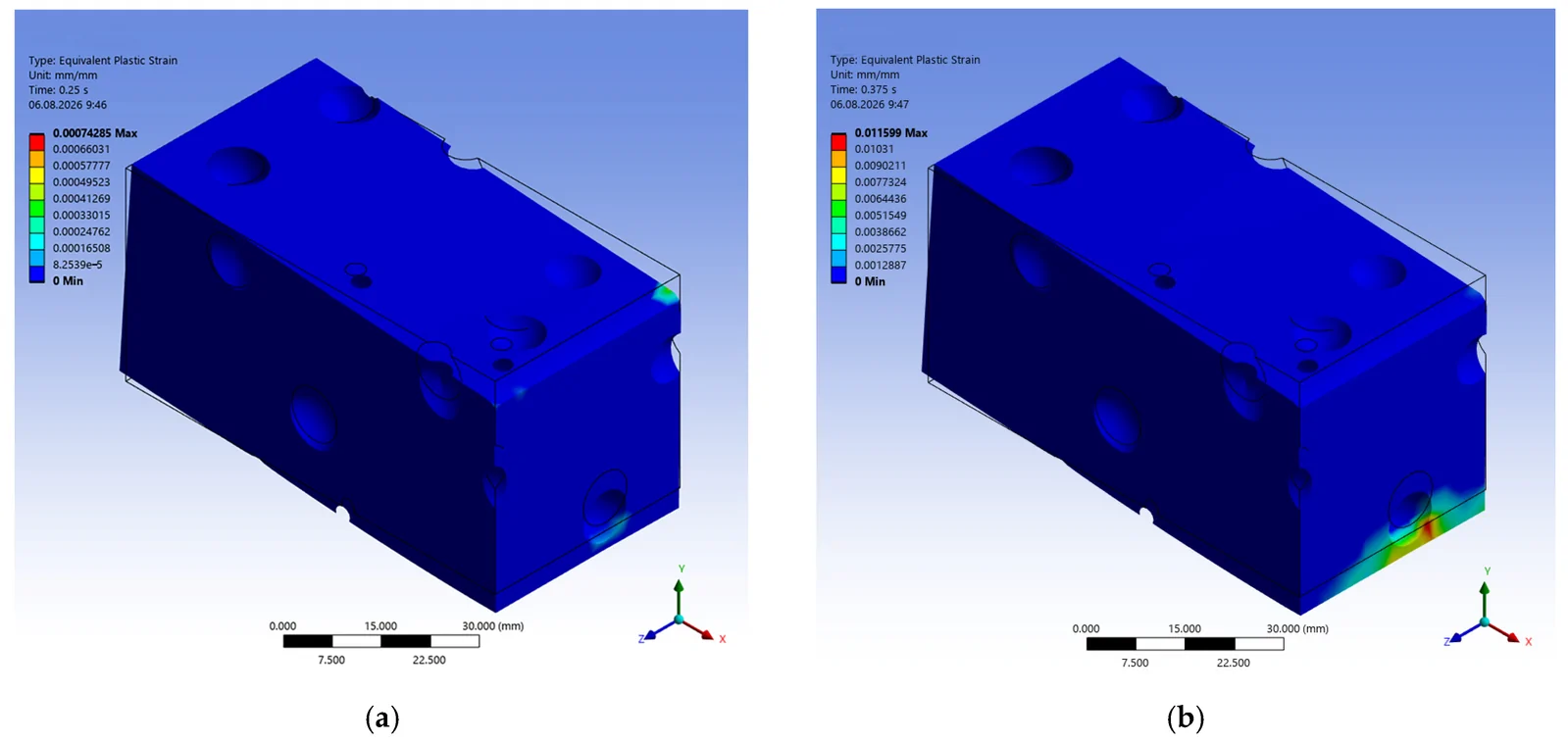

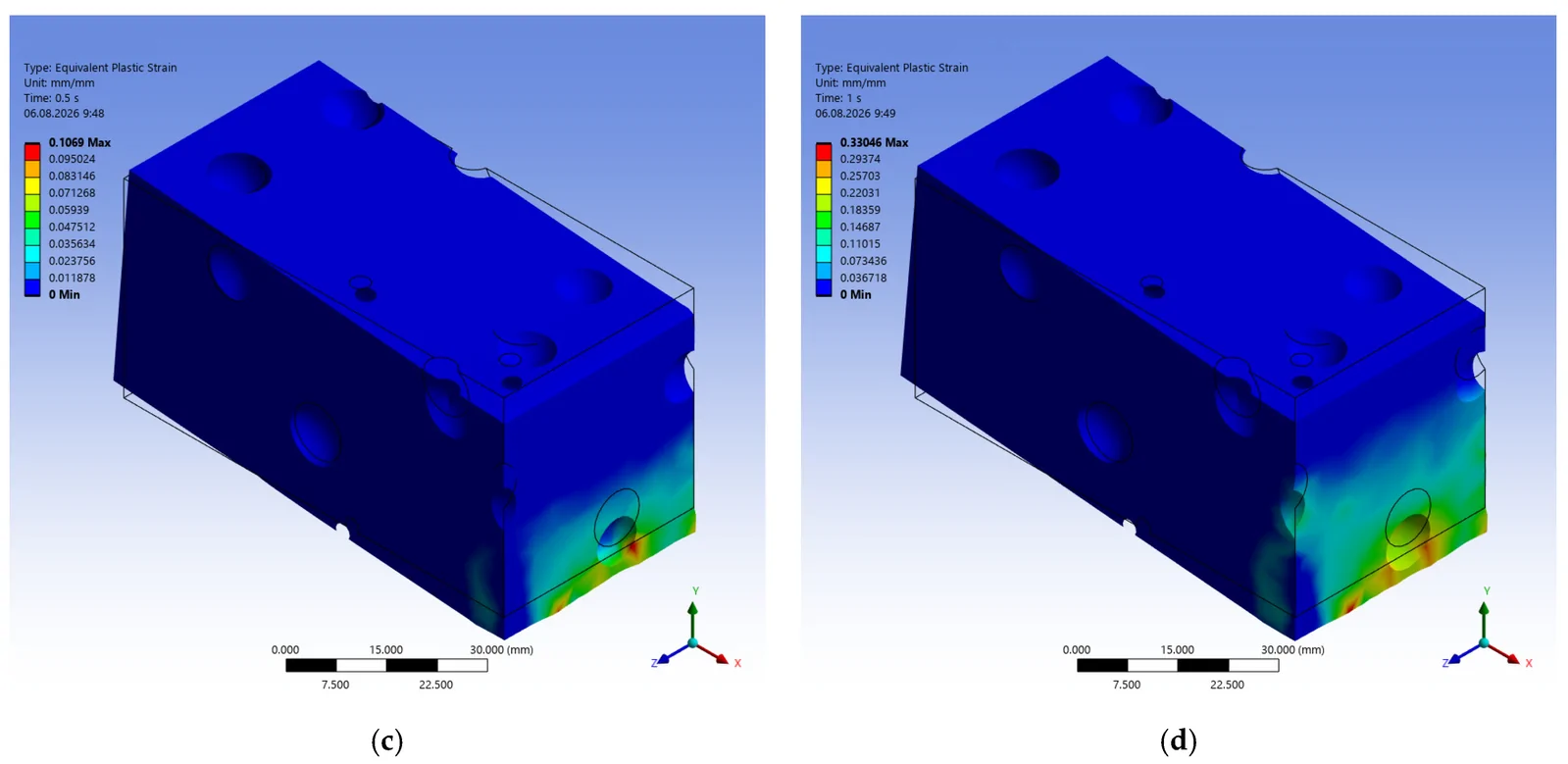
Sequential development of plastic deformation: (**a**) at 25% of the maximum load; (**b**) at 37.5% of the maximum load; (**c**) at 50% of the maximum load; and (**d**) at 100% of the maximum load.

**Figure 16:**
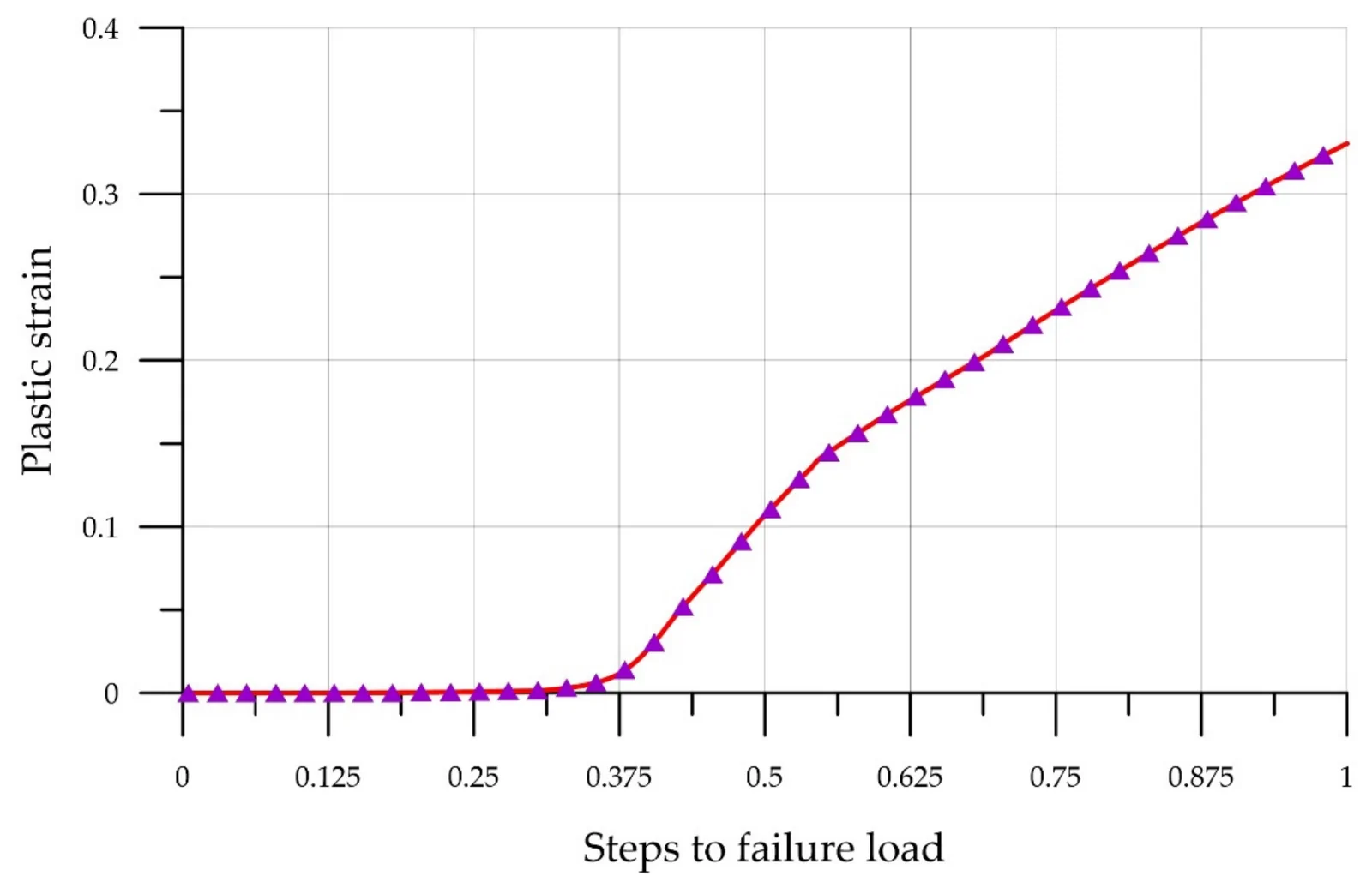
Plastic deformation of a composite with polypropylene spheres as a function of the relative loading steps (dimensionless units).

The sequential progression of plastic deformation (cracks) in a foam concrete composite containing polypropylene spheres is depicted in Figure 15 and Figure 16.

Figure 16 shows that plastic deformation develops in several stages. Up to 25% of the maximum load, the material is in the elastic deformation stage. Figure 15b and Figure 16 show that crack formation begins at approximately 37.5% of the maximum load. The crack initiates on the lower (expanded) flange, in the zone weakened by the sphere. Clearly, further crack propagation gradually shifts toward the upper flange; consequently, the crack grows, covering a larger area both vertically and longitudinally (the second crack). Figure 2 illustrates the crack’s path along the phase boundary, rather than through the spheres.

**Figure 17:**
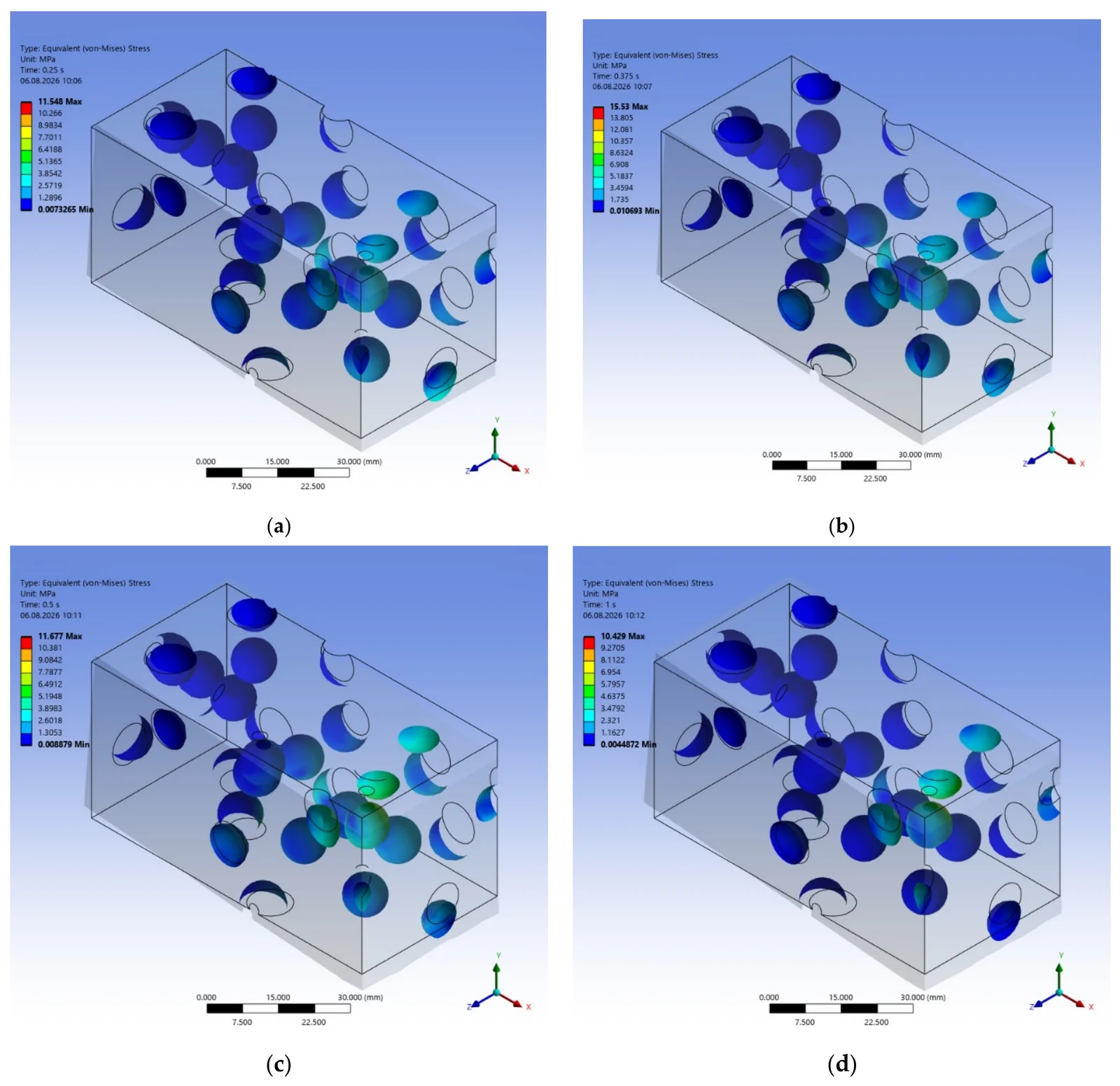
Stress–strain state development of polypropylene spheres: (**a**) at 25% of maximum load; (**b**) at 37.5% of maximum load; (**c**) at 50% of maximum load; and (**d**) at 100% of maximum load.

The stress–strain state of the composite’s spheres is presented in Figure 17.

Figure 17 shows that the spheres are subject to uneven stress–strain states. In the load-applied zone, von Mises stresses reach 10.4 MPa, which is significantly lower than the stresses arising under compression [11]. This is logically understandable, since significant stresses arise in the upper chord, where the spheres experience compressive stresses. In the tension zone, the spheres do not work, which leads to a reduction in the load-bearing capacity of the composite beam.

### 3.6. Discussion

The review of existing research shows that studies devoted to identifying the relationship between hollow polymer spheres and the flexural strength of foam concrete are limited. The most compelling evidence in the presented studies is derived from density and compressive behavior data rather than flexural testing [61,62,63,64,65]. Hollow spheres are described as controlled, low-density particles suitable for forming the isolated pore structure of foamed concrete and their addition typically reduces density while altering the pore network [66,67]. In similar cementitious systems, smaller or better bonded hollow inclusions typically improve compressive performance, while larger or poorly bonded inclusions can weaken it [68]. A promising avenue of research is the application of machine learning methods [69] to predict the properties of foamed concrete. However, this is currently hampered by the lack of a representative dataset.

This article demonstrates the mechanism by which hollow spheres influence the flexural behavior of a composite. Hollow polymer spheres reduce flexural strength if they create more voids or weak interphase boundaries, as evidence has linked higher porosity and interphase defects with poorer mechanical performance in foamed concrete–cement materials [70]. A counter-effect is also possible: if the spheres are small, well distributed, and well bonded, they may improve the pore structure and overall mechanical behavior. However, existing studies mainly demonstrate this for compressive rather than flexural response. Work on flexural behavior of foamed concrete materials is limited, reporting increased flexural strength for fibers and other fillers but no direct results on flexural strength for the hollow polymer spheres themselves [71,72]. Hollow polymer spheres likely act as lightweight pore-forming inclusions in foamed concrete, so their main documented effect is density reduction, with mixed or negative effects under compression depending on size and cohesion. As for the bending strength, the data provided is indirect and suggests that it may decrease if the spheres are not very well dispersed.

## 4. Conclusions

This research focuses on the numerical simulation of the thermophysical characteristics of structural foam concrete containing hollow polymer spheres. The subsequent key findings are as follows:

- An FEM model was created in the Material Designer module to predict the thermophysical properties of a composite made from structural foam concrete with embedded polymer spheres. The dependence of density, thermal conductivity, and specific heat on the diameter and properties of the polymer spheres were obtained. It should be noted that results for polypropylene were obtained theoretically and experimentally. For other polymers, these were obtained only theoretically.
- An investigation of the behavior of structural foam concrete with spheres in a non-uniform temperature field simulating the temperature effects of the environment in Arctic conditions was conducted. It was shown that the maximum equivalent von Mises stress was 3.3 MPa, indicating that the material operates in the elastic zone, but close to crack formation. The maximum displacement due to temperature differences was 0.087 mm, and the tensile stress in the longitudinal direction was *σ_X_* = 0.18 MPa.
- An FEM model was developed, and the stress–strain state analysis of structural foam concrete with polymer spheres under bending was conducted. The model is based on the Menetrey–Willam nonlinear deformation model of concrete. Verification of the model using load–displacement curves for various sphere sizes showed acceptable accuracy (up to 10%) in predicting strength properties. A significant discrepancy between theoretical and experimental data occurs in the zone of the descending branch during softening.
- Patterns of failure of structural foam concrete specimens with polymer spheres under bending were identified. It was shown that cracking begins in the lower chord of the tensile zone, weakened by the presence of the spheres. Composite failure is brittle, caused by the weakening of the interphase barriers. Since foam concrete has higher porosity and interphase defects with weaker mechanical properties than foam concrete-cement materials, the flexural strength of the composite decreases.

The value of this study lies in the fact that, using a regularly shaped filler in the form of polymer spheres, a numerical model was developed for further calculations, allowing for the simulation of the behavior of floating platform structures under temperature fluctuations and bending stresses.

This study demonstrates fundamentally new approaches to foam concrete, which will be subjected to bending loads under complex operational conditions. In particular, a variant of a complex combination of complex loads, including thermal and mechanical loads, was considered, taking into account hollow microspheres, which act as an additional reinforcer and blowing agent. Foam concrete itself, being a porous material, has a unique set of properties associated with the presence of air pores. In this case, the spheres act as an ultra-lightweight filler, also filled with air. This creates a simultaneously strengthening and porosity-forming component embedded within the porous matrix.

## Figures and Tables

**Table 1:** Foam concrete mixture formulation.

Consumption of raw materials per 1 m^3^	PC (kg/m^3^)	QS (kg/m^3^)	Water (L/m^3^)	R (kg/m^3^)
	390	560	170	8

**Table 2:** Experimental compositions of structural foam concrete with hollow polypropylene spherical fillers.

Component	Type of Composition					
	Control Composition	10 PS	12 PS	15 PS	19 PS	20 PS
Foam Concrete Mix (m^3^/m^3^)	1	0.85	0.85	0.85	0.85	0.85
PS (m^3^/m^3^)	–	0.15	0.15	0.15	0.15	0.15
PS Diameter (mm)	–	10	12	15	19	20

**Table 3:** Properties of samples of structural foam concrete with hollow polypropylene spherical fillers of different diameters.

Property	Type of Composition					
	Control Composition	10 PS	12 PS	15 PS	19 PS	20 PS
*ρ* (kg/m^3^)	1945	1809	1816	1840	1820	1855
*R* (MPa)	21.4	19.1	16.3	18.2	20.2	18.1
*R*_bt_ (MPa)	4.68	4.28	5.80	5.01	5.27	3.00

**Table 4:** Statistical characteristics.

Property and Statistical Characteristics	Type of Composition					
	Control Composition	10 PS	12 PS	15 PS	19 PS	20 PS
Density standard deviation (*S*_md_)	3.55	5.33	4.14	4.14	2.96	9.47
Density variation (*V*_md_, %)	0.18	0.29	0.23	0.23	0.16	0.51
Compressive strength standard deviation (*S*_m_)	1.23	0.91	1.15	1.15	0.95	0.71
Compressive strength variation (*V*_mR_, %)	5.73	4.76	7.03	6.31	4.70	3.92
Flexural strength standard deviation (*S*_mRbt_)	0.12	0.14	0.14	0.14	0.05	0.12
Flexural strength variation (*V*_mRbt_, %)	2.66	3.18	2.45	2.83	1.01	3.94

**Table 5:** Results of analysis of variance.

Source of Variability	SS	DF	MS	F	F_(0.05)_	*p*	Conclusion
Density							
Between samples	208.44	2	104.22	22.88	4.10	<0.001	Significant
Between the mixture compositions	38,432.44	5	7686.49				
Residual error	45.56	10	4.56				
General	38,686.44	17	–				
Compressive Strength							
Between samples	20.10	5	4.02	23.61	2.60	<0.001	Significant
Between the mixture compositions	94.92	5	18.98				
Residual error	4.26	25	0.17				
General	119.28	35	–				
Flexural Strength							
Between samples	0.13	2	0.06	44.13	4.10	<0.001	Significant
Between the mixture compositions	14.03	5	2.81				
Residual error	0.01	10	0.00				
General	14.17	17	–				

**Table 6:** Properties of the source materials.

Type of Material	Density (kg/m^3^)	Isotropic Thermal Conductivity Coefficient (W/(m × °C))	Specific Heat at Constant Pressure (J/(kg × °C))	Coefficient of Thermal Expansion (1/°C)
Foam concrete	1945	1.8	780	1.25 × 10^−5^
Polypropylene	950	0.2	2300	11.0 × 10^−5^
Polyethylene	960	0.4	2300	15.0 × 10^−5^
Polyamide	1400	0.25	1150	0.98 × 10^−5^

**Table 7:** Density of the composite with polymer spheres.

Material of Polymer Spheres	Diameter of Polymer Spheres (mm)				
	10	12	15	19	20
Polyethylene	1666.5	1665.8	1665.0	1664.4	1664.1
Polyamide	1668.7	1667.6	1666.4	1665.8	1665.2
Polypropylene	1666.3	1665.6	1664.8	1664.0	1663.9

**Table 8:** Menetrey–Willam model parameters for a lightweight concrete matrix.

Title	Units	Value
Young’s modulus	MPa	1500
Poisson’s ratio		0.18
Bulk modulus	MPa	781.2
Shear modulus	MPa	635.6
Uniaxial compressive strength	MPa	26.0
Uniaxial tensile strength	MPa	3.8
Biaxial compressive strength	MPa	40.0
Ratio of initial biaxial compressive strength to uniaxial compressive strength	-	1.538
Dilatancy angle	degree	10
Plastic strength at uniaxial compressive strength		0.003867
Plastic strain at transition from power law to exponential softening		0.004067
Relative stress at the start of nonlinear softening		0.5
Residual relative stress at transition from power law to exponential softening		0.9
Residual compressive relative stress		0.08
Mode 1 area specific fracture energy	N/m	100
Residual tensile relative stress		0.05

**Table 9:** Properties of polymeric materials.

Property	Polypropylene	Polyamide	Polyethylene
Young’s modulus, GPa	1.5	2.7	1.3
Poisson’s ratio	0.33	0.41	0.316
Bulk modulus, GPa	1.470	5.55	2.26
Shear modulus, GPa	0.564	1.06	0.95
Yield strength, MPa	25	65	30
Tangent modulus, MPa	0.1	0.1	0.1

**Table 10:** The errors in modeling the peak load and the displacement corresponding to the peak load.

*D*, mm	$F_{err}$, %	$Eps_{err}$, %
10	−1.0929	−2.5
12	−10.5263	−12.7
15	0.947867	2.9
19.05	−11.7647	−12.8
20	−15.1515	−16.5

## Data Availability

The original contributions presented in the study are included in the article; further inquiries can be directed to the corresponding author.

## Abbreviations

The following abbreviations are used in this manuscript:

SSS	Stress–strain state
TC	Thermal conductivity
FC	Foam concrete
MWM	Menetrey–Willam model
PES	Polyethylene spheres
PIS	Polyamide spheres
PPS	Polypropylene spheres
